# Establishment and Validation of a Peroxisome-related Gene Signature for Prognostic Prediction and Immune Distinction in Hepatocellular Carcinoma

**DOI:** 10.7150/jca.65080

**Published:** 2022-02-28

**Authors:** Dan Miao, Qian Xu, Yuan Zeng, Rui Zhao, Xian Song, Zhuoyan Chen, Liuwei Zeng, Luying Zhao, Zhuo Lin, Fujun Yu

**Affiliations:** 1 Department of Gastroenterology, the First Affiliated Hospital of Wenzhou Medical University, Wenzhou, Zhejiang, China.; 2 Laboratory Animal Centre, the First Affiliated Hospital of Wenzhou Medical University, Wenzhou, Zhejiang, China.

**Keywords:** Hepatocellular carcinoma, Peroxisome, Gene signature, Overall survival, Immune status, Tumor microenvironment

## Abstract

**Background:** Hepatocellular carcinoma (HCC) is a highly heterogeneous disease, which makes the prognostic prediction challenging. Abnormal peroxisomes can promote the development of cancers. This study aimed to construct a prognostic model based on peroxisome-related genes and identify its prognostic prediction and immune distinction abilities in HCC.

**Methods:** The prognostic model was constructed based on The Cancer Genome Atlas (TCGA) and The International Cancer Genome Consortium (ICGC). Kaplan-Meier curve, time-dependent receiver operating characteristic curve and Cox analysis were used to evaluate the model. The immune status, tumor microenvironment, drug sensitivity and expression levels of the mRNA and protein between HCC and adjacent non-tumorous tissues were analyzed and compared.

**Results:** A prognostic model of 9 peroxisome-related genes was established and validated. Overall survival was markedly higher in the low-risk group relative to the high-risk group. The risk score was an independent prognostic factor. Tumor-related pathways were enriched in the high-risk group and the HCC patients in high-risk group showed depleted immune status. Furthermore, immune checkpoint-related genes, cell cycle-related genes, and multidrug resistance-related genes were overexpressed in the high-risk group. The expression levels of prognostic genes were negatively related to the anti-tumor drugs sensitivity. In addition, the expression level of each prognostic gene in HCC tissues was higher than that in adjacent non-tumorous tissues in an independent sample cohort and the similar results were found in most cancer types.

**Conclusion:** A signature based on the nine peroxisome-related genes is a promising biomarker of HCC and is beneficial to the realization of individualized treatment.

## Introduction

Hepatocellular carcinoma (HCC), the most common form of liver cancer, is among the highly prevalent types of malignancy all over the world and ranks the third cause of cancer-related death 1. In recent years, the mortality and occurrence rate of HCC has been reported to be increased 2. Because of the rapid development and metastasis, the prognosis of patients with HCC is poor 3, HCC patients usually diagnose at an advanced stage 4, and the 5-year survival rate is as low as 30% 5, 6. With the rapid development of gene sequencing technology, we have a deeper understanding of the molecular pathogenesis of HCC 7, 8. High-throughput analysis of a large number of samples shows that accumulated data can be used to identify key biomarkers related to HCC progression 9. However, the number of biomarkers associated with HCC prognosis is still limited. Therefore, it's an urgent need to explore a novel method to guide clinical treatments and improve the prognosis of HCC.

The peroxisome is a monolayer membrane organelle, which exists in all kinds of eukaryotic cells and mediates varieties of biological processes. Peroxisome has been considered an important site for the generation and removal of free radicals in cells 10. The balance mechanism of free radical generation and scavenging in the peroxisome is essential to maintain the normal function of cells 11, 12. When the peroxisome undergoes oxidative stress, free radicals will be produced, which are closely associated with the occurrence and development of human tumors, and tumor progression may be influenced in multiple aspects 13-15. Moreover, reduced or disappeared peroxisome was found in lung cancer and liver cancer cells 16, and changes in the oxidative stress pathway of peroxisomes in tumor cells play a very important role in mediating the development of liver tumors 13. Therefore, an in-depth understanding of the peroxisome process in HCC could provide an important solution for the development of a new treatment method. In recent years, gene chips and high-throughput sequencing technology have made great progress, which implies that the genetic signature of the peroxisome can be used to predict the overall survival (OS) of HCC.

In this present study, a 9-gene risk model of HCC with a good performance in prognostic prediction was established and it was validated by the external validation cohort. The model was further evaluated under various clinical settings including survival, clinical-pathological characteristics, immune infiltration, immune pathways, immune checkpoints, multidrug resistance-related genes and chemotherapy. Besides, the expression of prognostic genes between HCC and adjacent normal tissues was validated in an independent sample cohort. The results showed that the model could be used as an independent prognostic evaluation index for HCC patients and could provide a new perspective for individual treatment for HCC patients.

## Materials and Methods

### Data collection

Transcriptome profiling data and related clinical data were downloaded from the Cancer Genome Atlas Liver Hepatocellular Carcinoma dataset (TCGA-LIHC, https://portal.gdc.cancer.gov/) as the train cohort. The International Cancer Genome Consortium Liver Hepatocellular Carcinoma dataset Japan (ICGC-LIRI-JP, https://dcc.icgc.org/) was regarded as the validation cohort. The downloaded profiles all comply with the TCGA and ICGC data access rules. The inclusion standard was that the patients pathologically diagnosed as hepatocellular carcinoma. The exclusion criteria were: 1) patients with hepatocellular metastasis and cholangiocarcinoma; 2) patients with co-existing cancers of other tissues; 3) Patients whose HCC samples lacked RNA sequencing data; 4) patients who lack time and status to survive; and 5) the follow-up with 0 day. A peroxisome-related gene set including 104 genes was retrieved from the Molecular Signatures Database (MsigDB, https://www.gsea-msigdb.org/gsea/index.jsp), **(Table S1)**.

### Identification of differentially expressed genes (DEGs) between HCC and adjacent non-tumorous tissues

The R package “limma” was used to identify DEGs between 365 HCC and adjacent non-tumorous tissues with the false discovery rate (FDR) < 0.05. Protein-protein interaction (PPI) networks of differently expressed peroxisome-related genes were constructed by using the STRING database.

### Establishment and validation of a peroxisome-related prognostic model

We firstly carried out univariate Cox regression analysis to find prognostic genes among the DEGs and identify the prognostic value of the DEGs for OS. Subsequently, the least absolute shrinkage selection operator (LASSO) Cox regression analysis was further performed to narrow down the number of candidate genes. The risk score was calculated by the following equation: Score =e sum (each gene's expression × corresponding coefficient). And all HCC patients were divided into high- and low-risk groups on account of the median value of risk score. To validate the reliability of this classification, we performed principal component analysis (PCA) and t-distributed stochastic neighbor embedding (t-SNE) to display the distribution of different groups using the "Rtsne" R package. The Kaplan-Meier curve analysis (log-rank test) was used to evaluate the survival difference between two groups via the "Survival" R package. The time-dependent receiver operating characteristic (ROC) curves were plotted, and the area under the curve (AUC) values were calculated with the application of the “SurvivalROC” R package to assess the predictive ability. Then, we further validated the predictive capacity of this model in the ICGC cohort.

### Identification of Independent prognostic factors for OS in HCC

Univariate and multivariate Cox regression analyses were performed to confirm whether the model could be independent of other clinical parameters (including gender, age, histological grade, and tumor stage) in predicting OS of HCC patients.

### Evaluation of immune status in different risk groups

We calculated the relative infiltrations of immune cell types and immune functional pathways by single sample gene set enrichment analysis (ssGSEA), analyzing tumor microenvironment (TME) in different risk groups. Six immune subtypes were defined to measure immune infiltrates in immune infiltration 17. The association between the risk score and immune infiltration subtypes in TME was analyzed by analysis of variance (ANOVA). Tumor stemness features extracted from HCC transcriptomic were measured by stemness score based on RNA methylation (RNAss) 18, and Wilcoxon test was carried out to analyze the RNAss in different risk groups.

### Immune checkpoint genes, cell cycle-related genes, multidrug resistance genes expression in different risk groups

Wilcoxon test was carried out to analyze the expression of immune checkpoints genes, cell cycle-related genes and multidrug resistance genes in different risk groups, respectively. Pearson correlation was used to analyze the correlation between the risk scores and the genes.

### GO and KEGG Functional Enrichment Analyses

To explore main biological functions and signaling pathways in different risk groups, Gene Ontology (GO) and Kyoto Encyclopedia of Genes and Genomes (KEGG) pathway enrichment analyses were conducted through gene set enrichment analysis (GSEA) in GSEA software 4.1. FDR < 0.05 was regarded as statistically significant.

### Anti-tumor drugs sensitivity analysis

The NCI-60 database was accessed through the CellMiner interface, which contains 60 different cancer cell lines from 9 different types of tumors (https://discover.nci.nih.gov/cellminer). Pearson correlation was used to analyze the correlation between the prognostic gene expression and the 263 anti-tumor drugs proved by the FDA or obtained from clinical trials. The 263 drugs were showed in **Table S2**.

### qRT-PCR analysis

The real-time quantitative-polymerase chain reaction (qRT-PCR) assays were used to validate the mRNA expression levels of the 9 prognostic genes screened above in 20 paired samples of HCC and adjacent non-tumorous tissues. The samples were from the First Affiliated Hospital of Wenzhou Medical University. And this study was approved by the Review of Ethics Committee in Clinical Research of the First Affiliated Hospital of Wenzhou Medical University. Written informed consents were obtained from all patients for the use of the biospecimens for research purposes. Total RNA was extracted with the Trizol reagent following the manufacturer's instructions (Servicebio). Then cDNA was synthesized using reverse transcriptase that provided by Thermo. RT-PCR analysis was performed using FastStart Universal SYBR Green Master (Roche) by ABI StepOne (Applied Biosystems). The primers used are shown in **Table S3**.

### Immunohistochemical analysis

The protein expression of prognostic peroxisome-related genes between HCC and adjacent non-tumorous tissues was evaluated in 10 paired samples by the Immunohistochemistry (IHC) method. Samples were fixed with 10% formalin, dehydrated with alcohol, xylene transparentized, embedded with paraffin immersion wax, and sectioned with paraffin microtome. The sections were dewaxed with conventional xylene and dehydrated with gradient alcohol. After the antigen was repaired, the endogenous peroxidase was blocked by H2O2 (3%) for 25 minutes, and the nonspecific bindings were blocked by 10% rabbit serum or 3% bovine serum albumin for 30 minutes. IHC staining was carried out for the protein expression of *ABCC5, BCL10, FDPS, ITGB1BP1, MSH2, PABPC1, PRDX1, SLC25A19,* and* YWHAH* using specific primary antibodies at 4 °C overnight, followed by staining with species-specific secondary antibodies labeled with horseradish peroxidase. The information of the antibodies was provided in **Table S4**. After the slices were stained with diaminobenzidine and counterstained with hematoxylin, the sample was dehydrated, transparent, sealed, observed, and photographed.

### Statistical analyses

All statistical analyses were performed in R software (Version 4.0.2). Gene expression levels between HCC and adjacent non-tumorous tissues were analyzed by the Wilcoxon test. Differences in proportions were assessed by the Chi-squared test. Kaplan-Meier analysis was used in different groups for OS. Univariate and multivariate Cox regression analyses were used to screen independent predictors for OS. Mann-Whitney test was used to compare the ssGSEA scores of immune cells or the activity immune pathways in different risk groups. Spearman correlation was carried out to analyze the associations between the risk score and immune checkpoint-related genes, cell cycle-related genes, and multidrug resistance-related genes. Pearson correlation was utilized to analyze the relationship of the prognostic gene expression to drug sensitivity. A two-tailed P < 0.05 was considered to be statistically significant.

## Results

**Figure 1:**
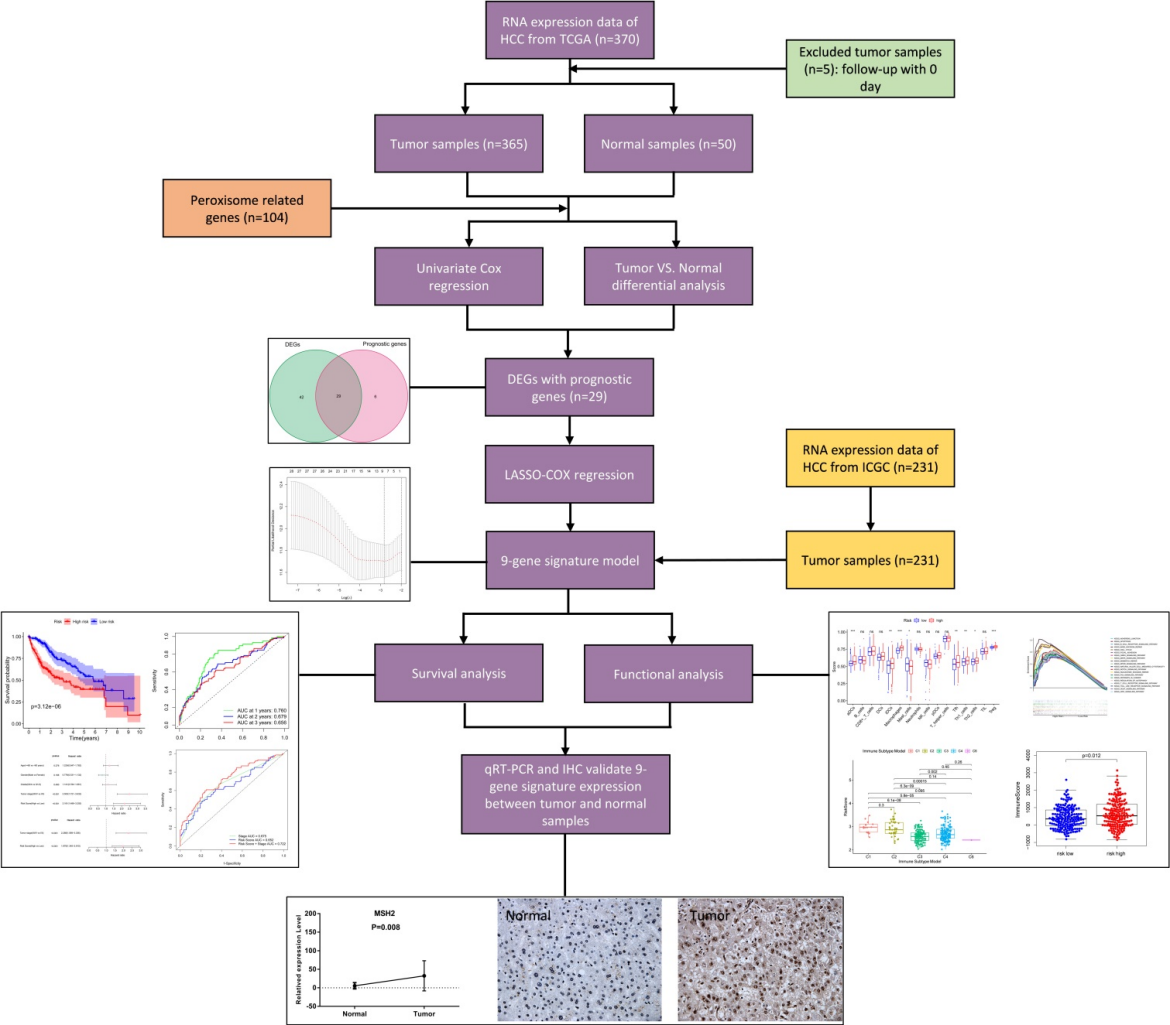
Flow chart of data collection, analysis and experiment.

**Figure 1** shows the process of establishing the gene signature and the prognostic model of this study. According to the criteria for the inclusion and exclusion of HCC patients, we ultimately identified and included 365 patients from TCGA dataset and 231 patients from ICGC dataset. The detailed clinical characteristics are summarized in **Table 1**.

### Identification of DEGs related to peroxisome in the TCGA cohort

**Figure 2:**
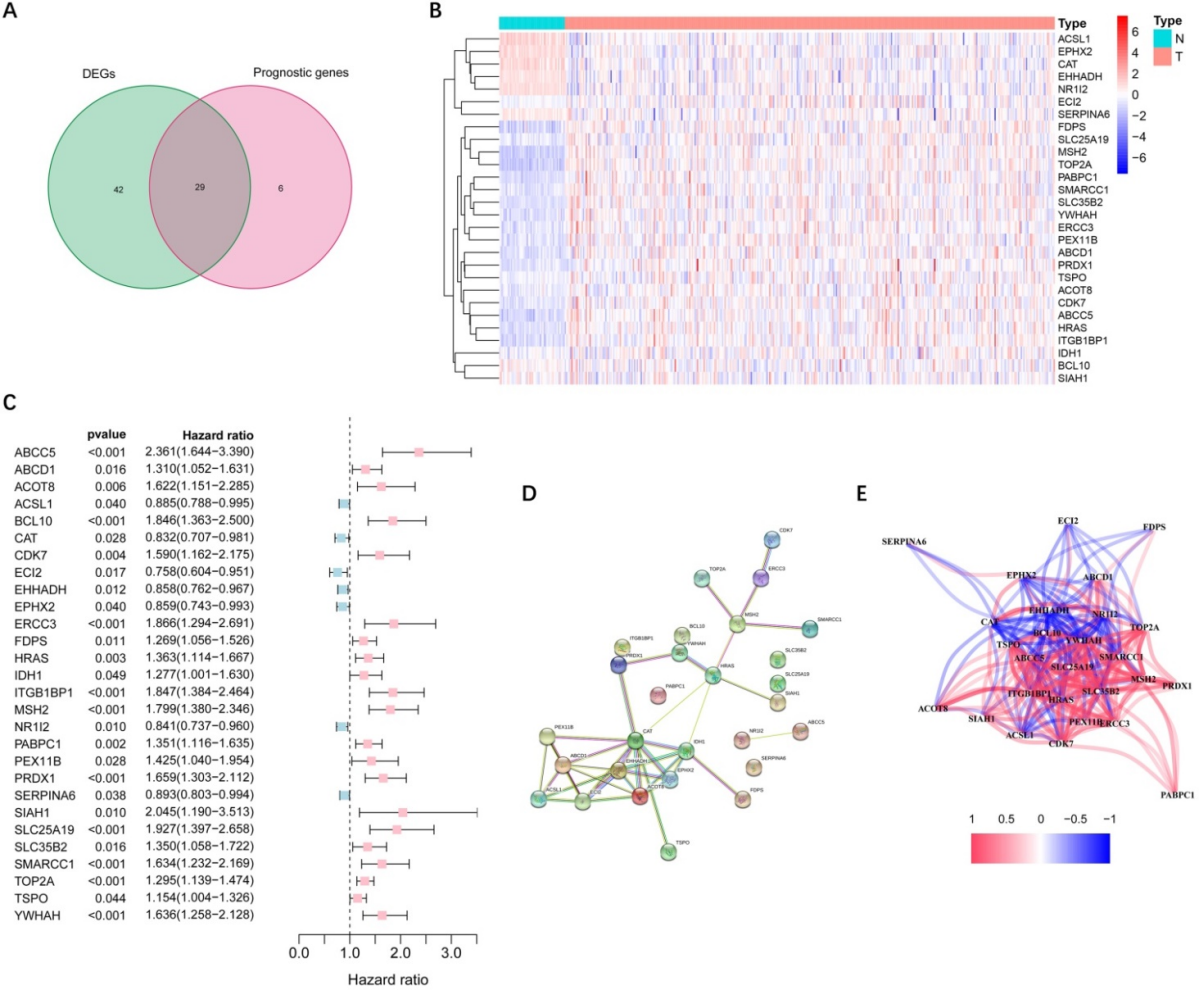
** Identification of candidate peroxisome-related genes in the TCGA cohort. (A)** Venn diagram to identify DEGs between HCC and adjacent normal tissues. **(B)** Expression of the 28 overlapping genes between HCC and adjacent normal tissues. **(C)** Univariate Cox regression analysis identifying prognostic variables with HR with 95% CI and P values. **(D)** The protein-protein interaction network of candidate genes. **(E)** The correlation network of candidate genes. DEGs, differentially expressed genes; TCGA, the Cancer Genome Atlas.

More than half of the 104 peroxisome-related genes (71/104, 68.3%) were differentially expressed between tumor and adjacent non-tumorous tissues. Univariate Cox regression analysis showed that 29 DEGs were related to OS **(Figure 2A)**. **Figure 2B-C** implies that 28 genes in the TCGA cohort had significantly prognostic relevance (excluded *FABP6* gene that was not expressed in most samples). The interactions among these candidate genes were shown in PPI and correlation network **(Figure 2D-E)**.

### Establishment of the prognostic model in the TCGA cohort

**Figure 3:**
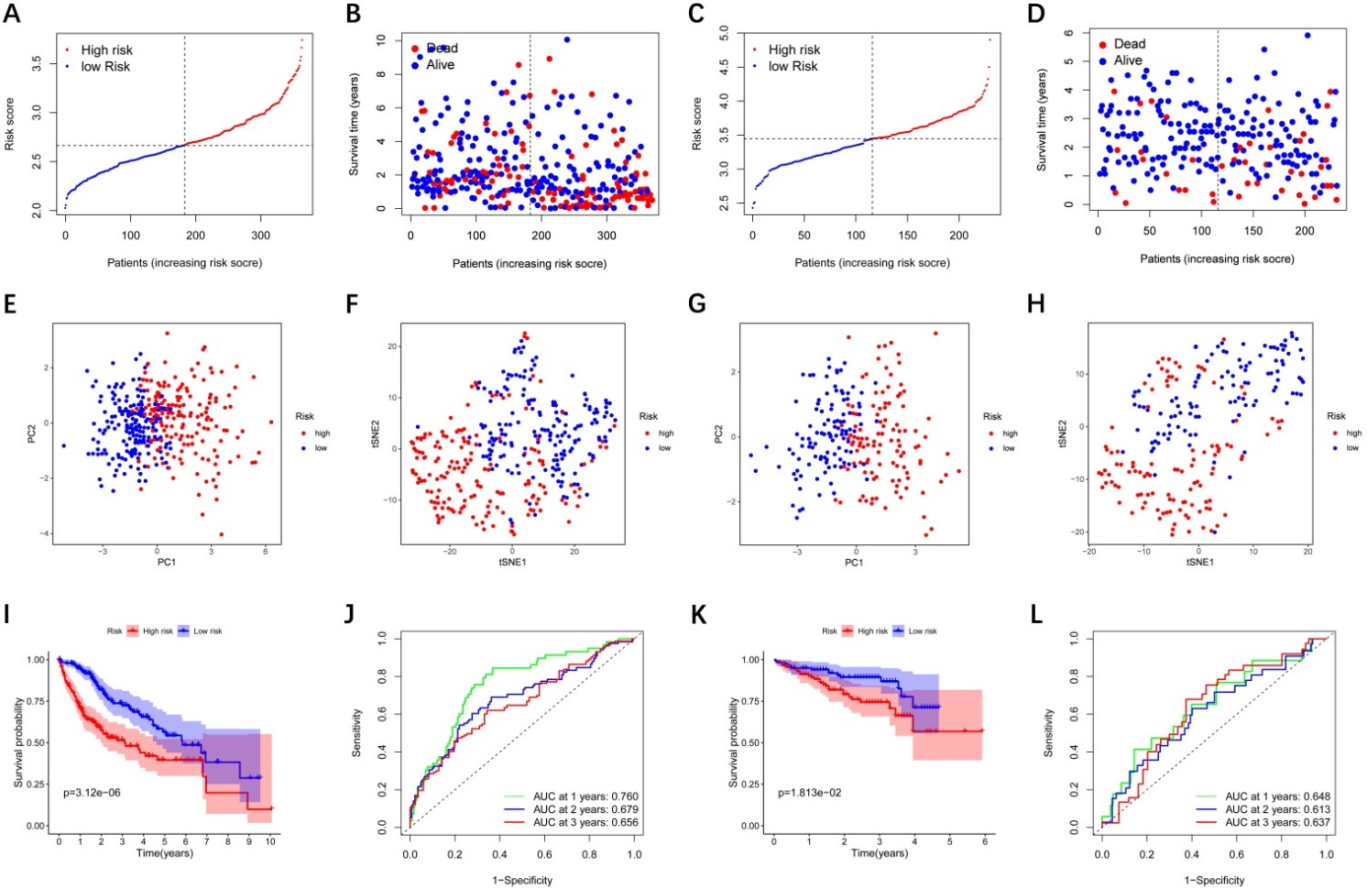
** Prognostic analysis of the 9-gene signature model in the TCGA cohort and ICGC cohort.** TCGA cohort (A, B, E, F, I, J), ICGC cohort (C, D, G, H, K, L). **(A, C)** The distribution and median value of the risk scores. **(B, D)** Distributions of the overall survival (OS) status. **(E, G)** PCA plot **(F, H)** t-SNE analysis **(I, K)** Kaplan-Meier curves for OS of patients in the high-risk group and low-risk group. **(J, L)** Time-dependent ROC curves for OS. ICGC, International Cancer Genome Consortium.

LASSO Cox regression analysis was performed to establish a prognostic model based on the above-mentioned 28 DEGs. Subsequently, a 9-gene signature was identified, consisting of *ABCC5, BCL10, FDPS, ITGB1BP1, MSH2, PABPC1, PRDX1, SLC25A19,* and* YWHAH*** (Figure S1)**. The risk score was calculated as follows: score = 0.238 * expression level of *ABCC5*+ 0.144 * expression level of *BCL10* + 0.022 * expression level of *FDPS* + 0.137 * expression level of* ITGB1BP1* + 0.026 * expression level of *MSH2* + 0.011 * expression level of* PABPC1* + 0.185 * expression level of *PRDX1* + 0.031 * expression level of *SLC25A19* +0.016 * expression level of *YWHAH*. The median risk score of the TCGA cohort served as the unified cut-off for dividing HCC patients into high- and low-risk groups **(Figure 3A)**. Moreover, we observed that the high-risk group had a significantly higher percentage of HCC patients with worse clinicopathological characteristics, such as an advanced tumor stage and a later histological grade **(Table 2)**. As shown in **Figure 3B**, HCC patients in the high-risk group presented a shorter survival time and more occurrences of death. PCA and t-SNE analyses showed that patients in the two groups were distributed in two different directions** (Figure 3E-F)**. Conformably, the Kaplan-Meier curve indicated that the prognosis of the high-risk group had a significantly shorter OS than that of the low-risk group (P<0.001)** (Figure 3I)**. Time-dependent ROC curves of the prognostic model at 1, 2, and 3 years were 0.760, 0.679, and 0.656, respectively, indicating a good predictive performance** (Figure 3J)**. According to the optimal cut-off expression value of each prognostic gene, survival analysis showed that the high expression levels of the 9 genes were all correlated to the poor prognosis (P<0.05)** (Figure S2A-I)**. Moreover, **Figure S3** implies that the expression of each prognostic gene in HCC was distinctly higher than that in adjacent non-tumorous tissues (P<0.001).

### External validation of the prognostic model in the ICGC cohort

To verify whether the prognostic model is robust, we used the independent cohort (ICGC) for external validation. Similar to the results of the TCGA cohort, HCC patients were stratified into a high-risk group and a low-risk group based on the median value from TCGA **(Figure 3C)**. And the high-risk group showed significantly poorer OS relative to the low-risk group** (Figure 3D)**. PCA and t-SNE analyses also indicated that patients in two groups were distributed in discrete directions** (Figure 3G-H)**. The Kaplan-Meier curve demonstrated that HCC patients in the high-risk group had a shorter survival time **(Figure 3K)**. In the ICGC cohort, time-dependent ROC curves at 1, 2, and 3 years were 0.648, 0.613, and 0.637, respectively **(Figure 3L)**.

### Independent prognostic value of the 9-gene signature

**Figure 4:**
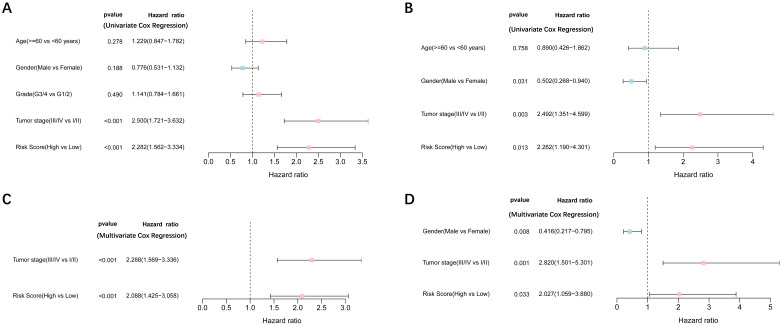
** Results of the univariate and multivariate Cox regression analyses regarding OS TCGA cohort** (A, C), ICGC cohort (B, D). **(A, B)** Univariate Cox regression analyses to screen OS-related factors. **(C, D)** Multivariate Cox regression analyses to screen OS-related factors.

**Figure 5:**
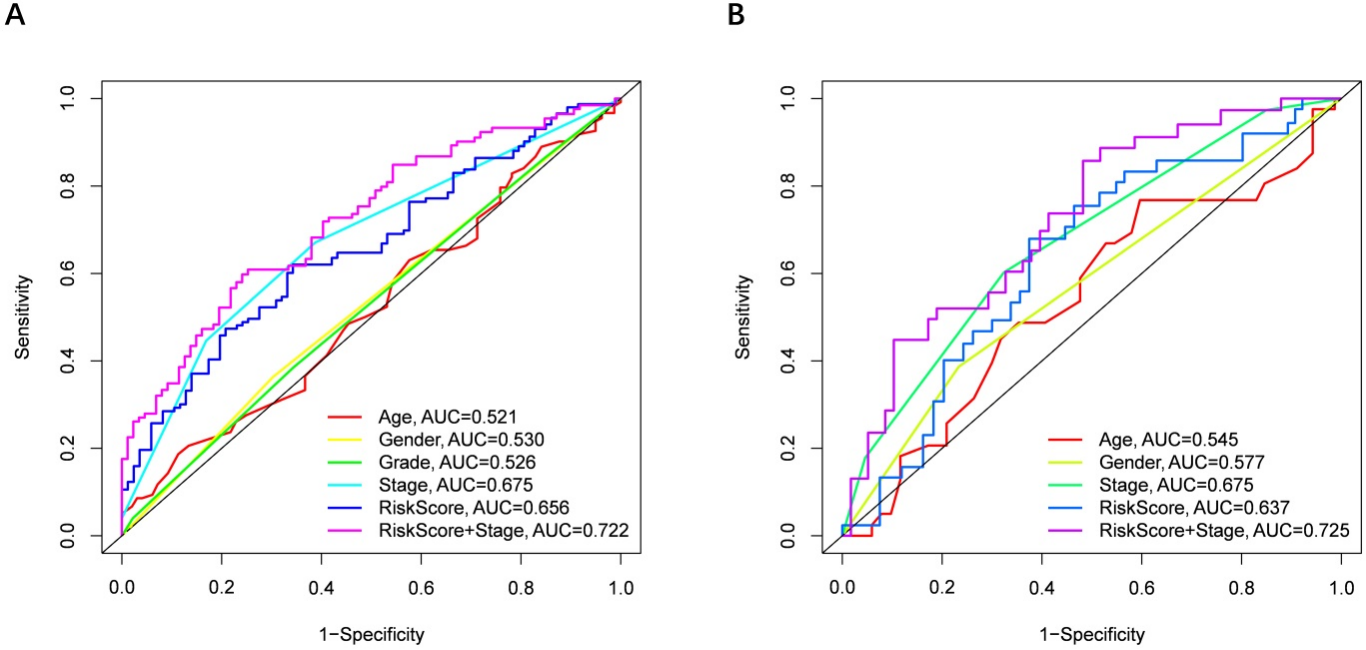
** The area under the curve (AUC) of clinical characteristics, risk score, and the risk score combined with tumor stage at 3-year OS. (A)** TCGA cohort, **(B)** ICGC cohort.

Univariate and multivariate Cox regression analyses were performed to examine the predictive capability of the prognostic model. As shown in **Figure 4A**, univariate Cox regression analysis revealed that the risk score and the tumor stage were significantly associated with OS of HCC (P < 0.001). Further, multivariate Cox regression analysis indicated that the risk score was an independent prognostic factor affecting long-term survival in the TGCA cohort. **Figure 5A** shows that the AUC of the risk score, the stage and the risk score combined with the tumor stage at 3-year OS is 0.675, 0.652 and 0.722, respectively. The results mentioned above showed that the combined model had better prediction accuracy for OS. The same prognostic significance of the risk score was verified using the data obtained from the ICGC cohort **(Figure 4B, 5B)**.

### Risk score and prognostic genes in different clinical characteristics groups

**Figure 6:**
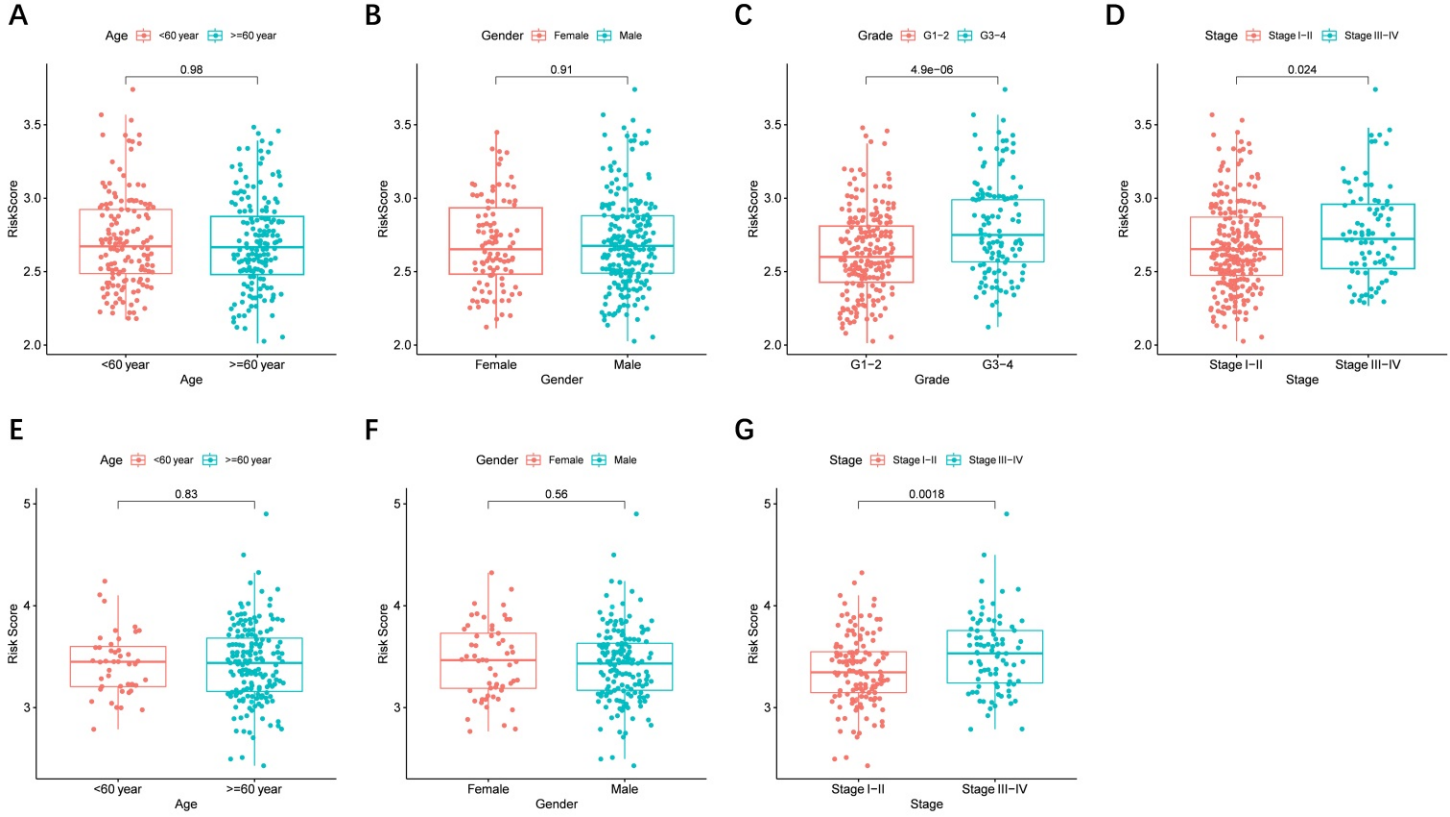
** The risk score in different groups stratified by clinical characteristics.** TCGA cohort (A-D), ICGC cohort (E-F). (**A, E**) Age, (**B, F**) gender, (**C**) tumor grade, (**D, G**) tumor stage.

The risk score distributed in different clinical characteristics groups (including gender, age, histological grade, and tumor stage) in HCC patients based on the TCGA and ICGC data is shown in** Figure 6A-G**, respectively. The risk score was higher in the high histological grade and advanced tumor stage in the TCGA cohort, and a similar conclusion was seen in the ICGC cohort (There were no data about the histological grade of LICH in the ICGC cohort). Furthermore, combining prognostic gene expression levels with clinical characteristics of HCC patients indicated that the expression of *MSH2* had significant differences in clinical characteristics **(Figure S4A-D)**.

### Relationship between risk score and immune status

**Figure 7:**
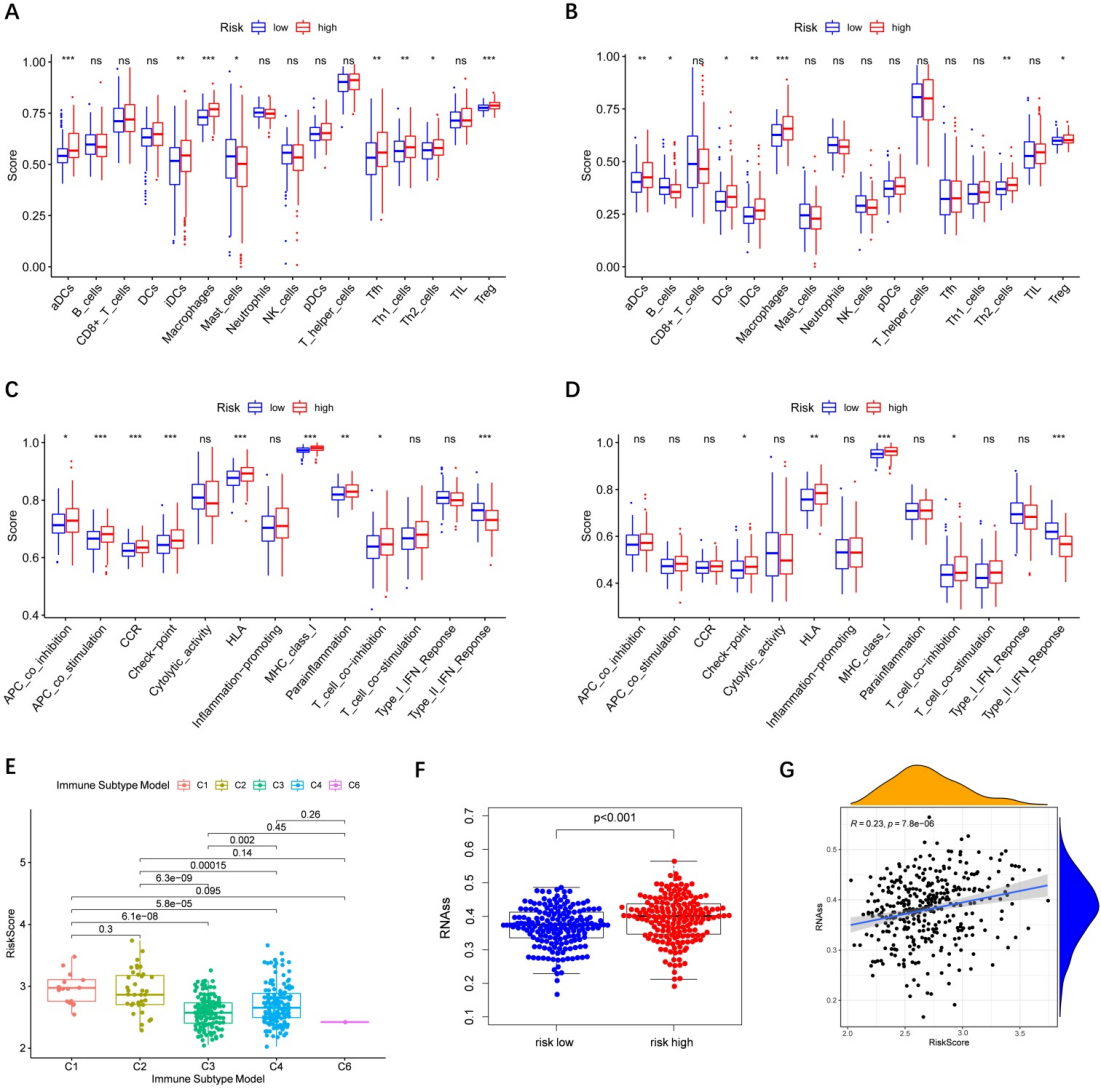
** Immune status between different risk groups and the association of risk score with RNAss.** TCGA cohort (A, C), ICGC cohort (B, D). **(A, B)** The scores of 16 immune cells. **(C, D)** The boxplots showing the 13 immune-related functions. **(E)** Comparison of the risk scores between different immune infiltrate subtypes. **(F)** The different scores of RNSss between HCC and adjacent normal tissues. **(G)** The relationship of risk score with RNAss. P values are shown as: ns, not significant; *, P < 0.05, ** P < 0.01, ***, P < 0.001.

Immune cells' infiltration in different risk groups was analyzed to investigate the impact of risk score on TME. In the high-risk group, the infiltration of aDCs, iDCs, Macrophages, and Th2 cells was significantly increased in the TCGA and ICGC cohorts (P< 0.01), implying that high expression of peroxisome-related genes may promote the release of these immune cells **(Figure 7A-B)**. In terms of immune function, we observed that type II IFN response in the low-risk group was significantly stronger than that in the high-risk group** (Figure 7C-D)**.

Subsequently, we estimated the risk score in different immune subtypes in HCC patients. Six immune subtypes defined to measure immune infiltrates in tumor immune response, numbered from lowest to the highest relative abundance of cytotoxic cells, were C1 (wound healing), C2 (INF-γ dominant), C3 (inflammatory), C4 (lymphocyte depleted), C5 (immunologically quiet), and C6 (TGF-β dominant) 19. No patient sample belonged to the C5 immune subtype in HCC. As shown in **Figure 7E**, the high-risk score was significantly correlated with C1 and C2, and the low-risk score was correlated with C3, C4, and C6. And the high levels of all the prognostic genes were positively related to the C1 and C2, showing their promoting role in the process of HCC. Correlations of the 9 prognostic genes with the 6 immune types were shown in **Figure S5**.

Tumor cells can gradually lose a differentiated phenotype and acquire progenitor and stem-cell-like features in the cancer progression 20. Study suggests that an abundant population of tumor cells with stemness features may be a signal of poor prognosis in HCC patients 21. Then the correlation between the risk model and tumor stemness measured by RNAss was explored. As shown in** Figure 7F-G**, RNAss was higher in the high-risk group than that in the low-risk group, and RNAss was positively with the risk score. What's more, we observed that prognostic genes had a positive association with RNAss except for *ABCC5, BCL10,* and* YWHAH* (P< 0.0001) **(Figure S6A)**. These results revealed that the high expression of prognostic genes in HCC correlated to increased cancer cell stemness was consistent with the fact that increased expression of prognostic genes supported worse survival.

**Figure 8:**
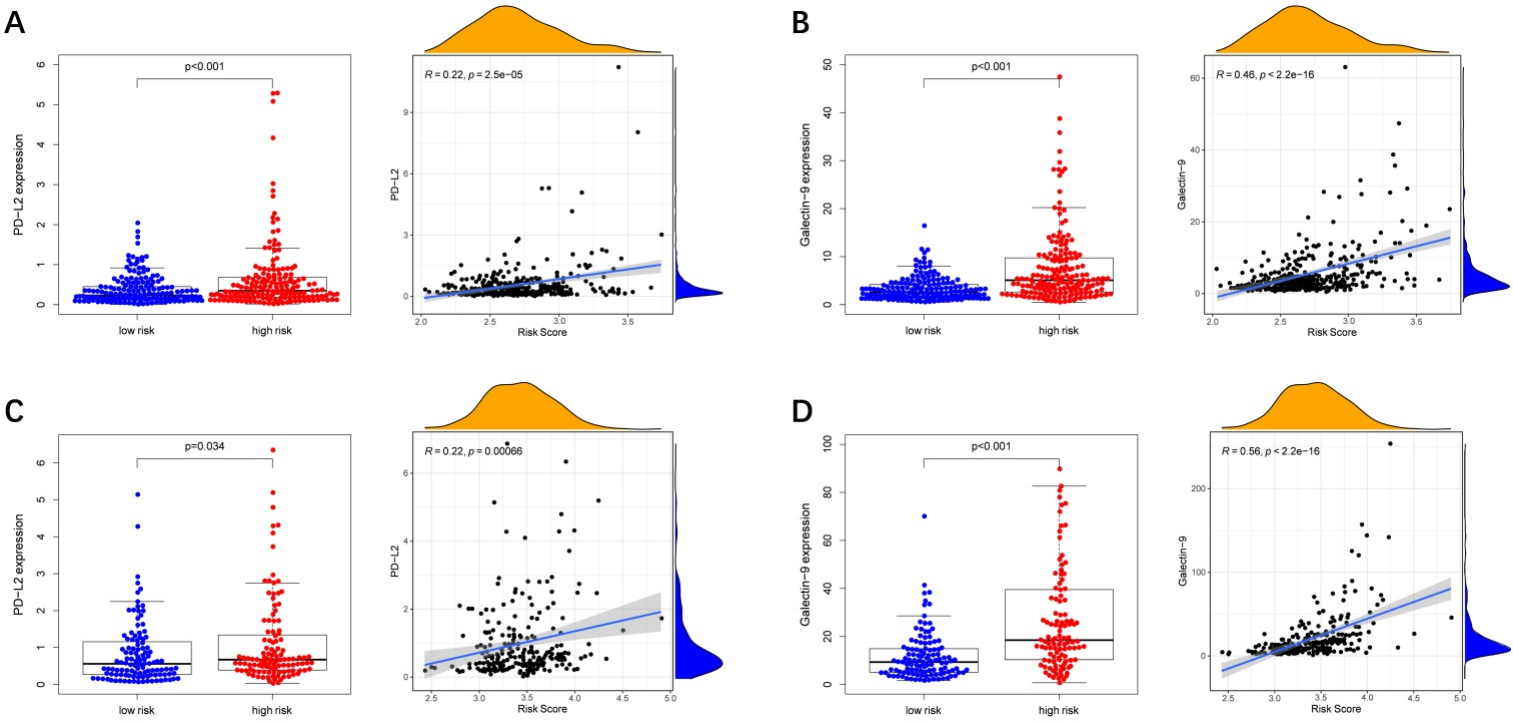
** Immune checkpoint genes expression in different risk groups and correlations of the immune checkpoint genes expression levels and the risk score.** (**A, B**) TCGA cohort, (**C, D**) ICGC cohort.

Immune checkpoint-associated genes play an important role in the process of cancer 20. We further explored correlations of the expression levels of immune checkpoint-associated genes in different risk groups. We found that *PD-L2* and *Galectin9* were overexpressed in the high-risk group compared with the low-risk group in the TCGA cohort **(Figure 8A-B)**. Furthermore, the expression levels of *PD-L2* and *Galectin9* were all positively correlated with the risk score in the TCGA cohort **(Figure 8A-B)**, implying that the prognostic model conducted in this study can distinguish the expression levels of the immune checkpoint-associated genes. What's more, the similar conclusion was shown in the ICGC cohort **(Figure 8C-D)**.

### GO and KEGG functional enrichment analysis

**Figure 9:**
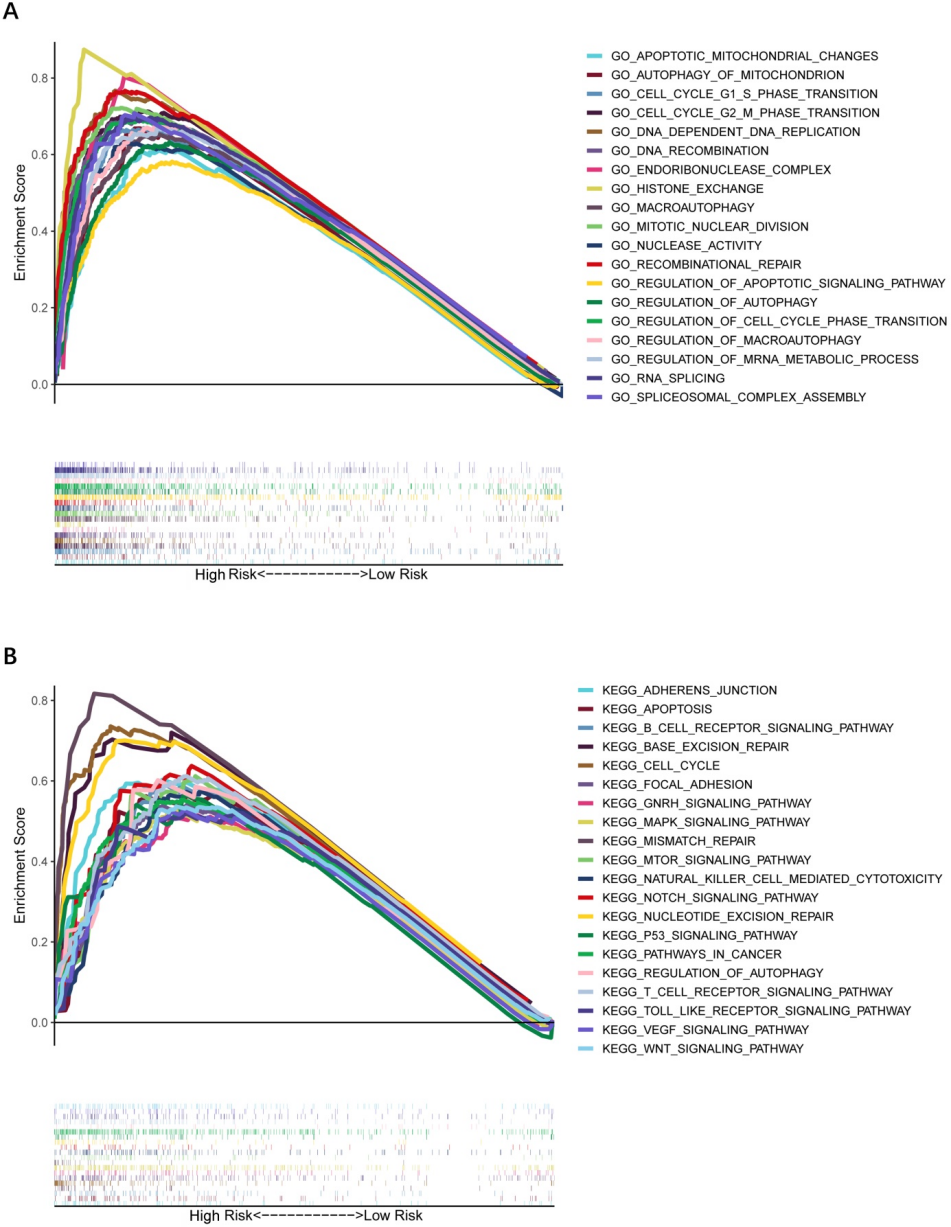
** Gene set enrichment analysis of biological function and pathway. (A)** GO, Gene Ontology. **(B)** KEGG, Kyoto Encylcopedia of Genes and Genomes.

**Figure 10:**
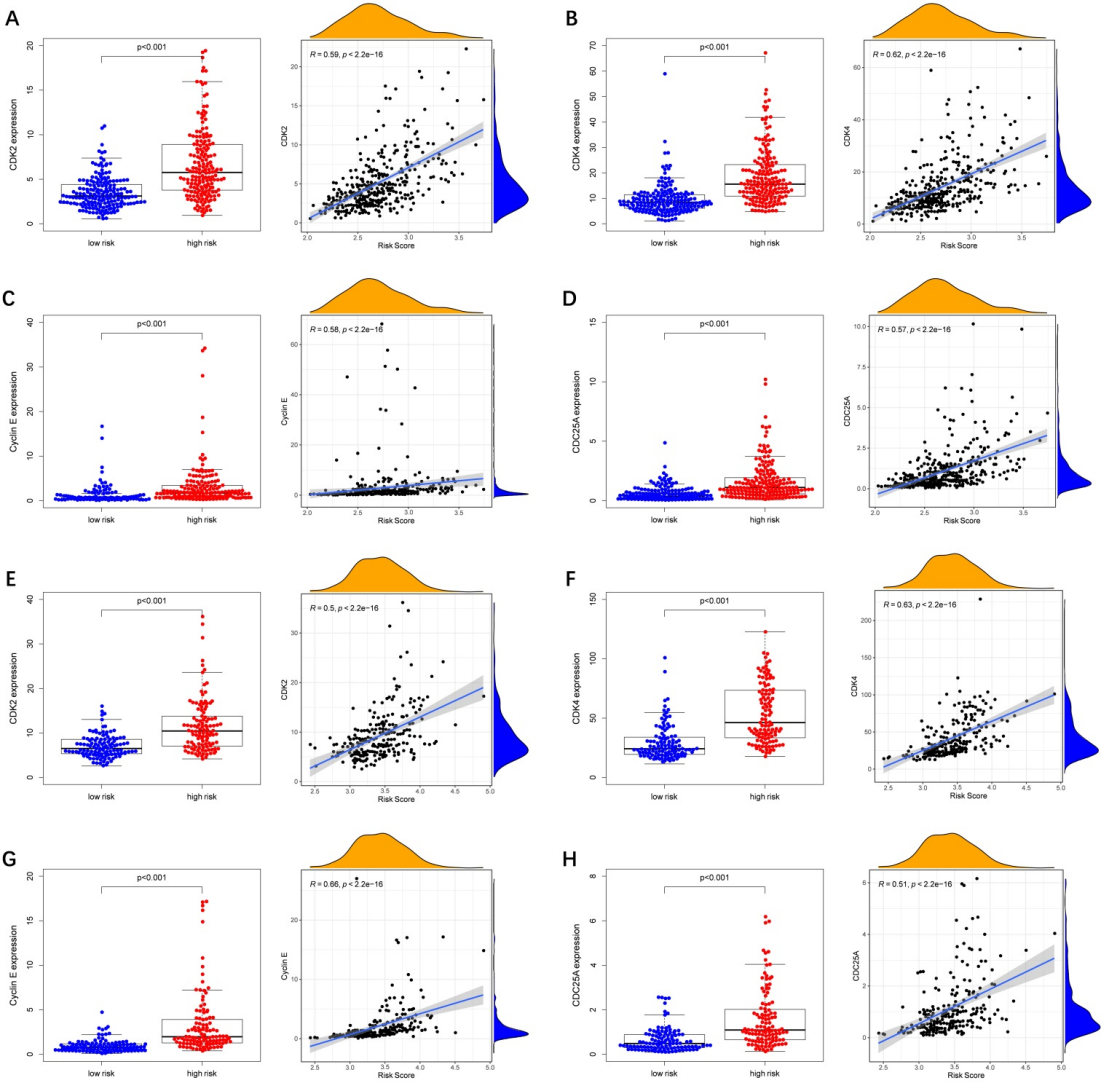
** Expression of cell cycle related genes in different risk groups.** (**A-D**) TCGA cohort, (**E-H**) ICGC cohort.

To further explore whether biological functions and pathways are correlated with the risk score signature, GO and KEGG were carried out in different groups in the TCGA cohort. We found that some cancer-related gene sets were significantly gathered in HCC patients with a high-risk score. As shown in **Figure 9**, significant functions associated with tumorigenesis were enriched in the high-risk group defined by the 9-gene signature with the FDR<0.05, including Cell cycle G1-S phase transition, Regulation of autophagy, Apoptotic mitochondrial changes, Focal adhesion, Cell cycle pathway, Erbb signaling pathway, Notch signaling pathway, P53 signaling pathway, Wnt signaling pathway and Pathways in cancer. The detailed information is displayed in **Figure S7-8**. We found that cell cycle related pathways were upregulated in the high risk group, so we further looked into cell cycle related genes in different groups. As shown in **Figure 10A-D**, CDK2, CDK4, Cyclin and CDC25A were highly expressed in the high risk group, and all of them were positively associated with the risk score in the TCGA cohort. The results in the ICGC cohort were consistent with the TCGA cohort** (Figure 10E-H)**.

### Analysis of the correlation between the risk model and multidrug resistance-related genes and chemotherapeutics

**Figure 11:**
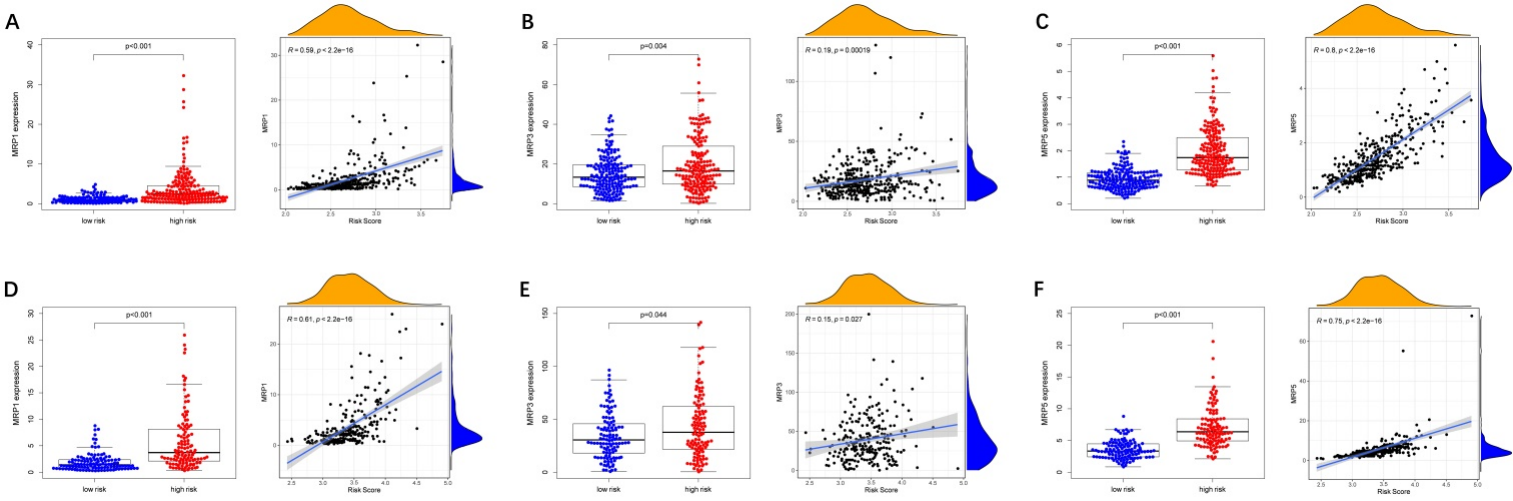
** Expression of anti-tumor drug genes in different risk groups.** (**A-C**) TCGA cohort, (**D-F**) ICGC cohort.

**Figure 12:**
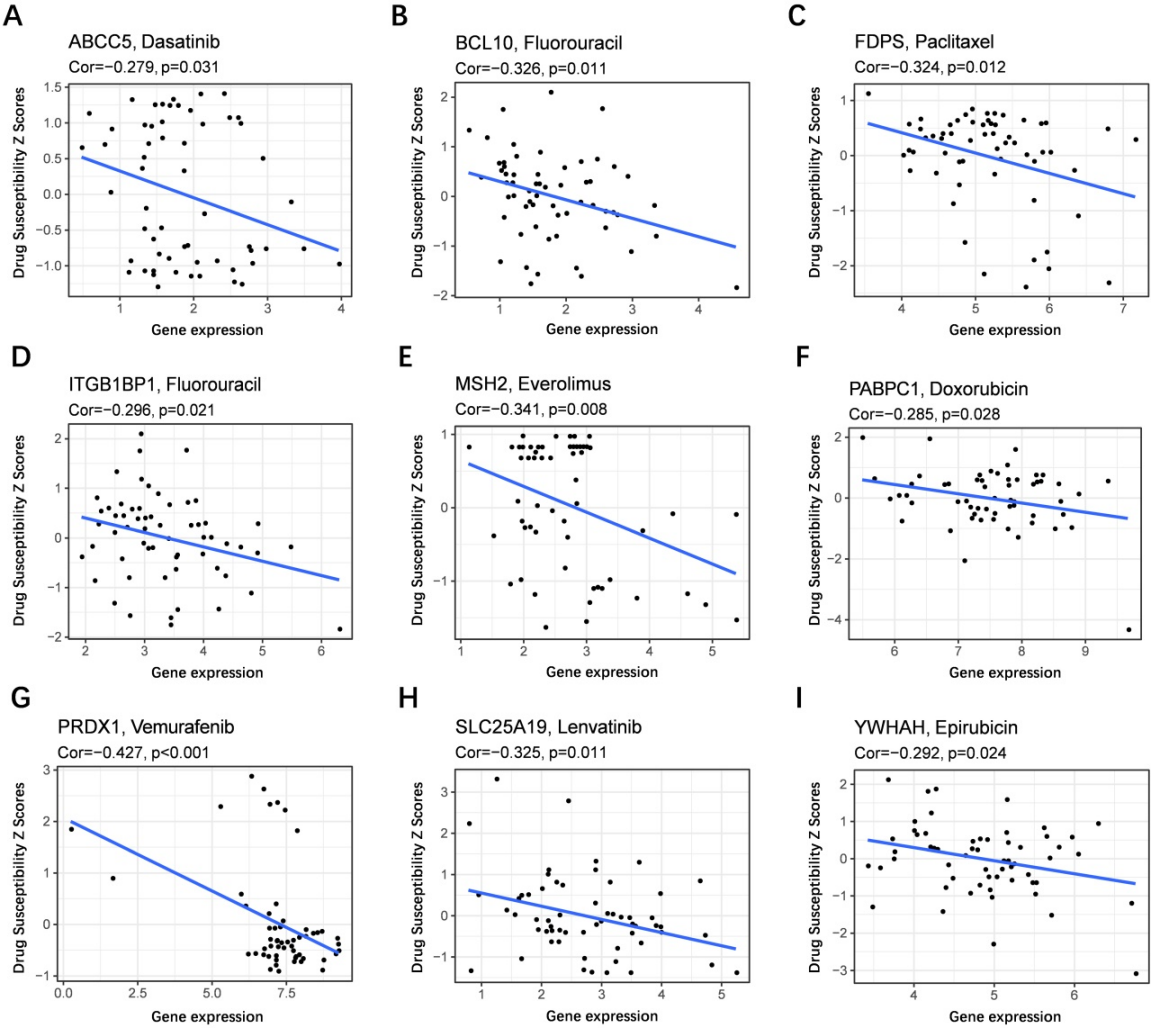
** Scatter plots of the association between the prognostic gene expression and anti-tumor drugs sensitivity.** (**A**) *ABCC5,* (**B**) *BCL10,* (**C**) *FDPS,* (**D**) *ITGB1BP1,* (**E**) *MSH2,* (**F**) *PABPC1,* (**G**) *PRDX,* (**H**) *SLC25A19,* (**I**) *YWHAH.*

To evaluate the model in the clinic for HCC treatment, we further explored correlations of the expression levels of multidrug resistance-related gens and the risk score in different risk groups. We found that the expression of *MRP1, MRP3* and *MRP5* were higher in the high-risk group than that in the low-risk group **(Figure 11A-C)**. Furthermore, the expression levels of *MRP1, MRP3* and* MRP5* were all positively correlated with the risk score **(Figure 11D-F)**, implying that the prognostic model conducted in this study can distinguish the expression levels of multidrug resistance-related genes. Based on the above findings, correlations of the risk score and sensitivity of antitumor drugs were performed and the results in **Figure 12** showed that the prognostic genes were negative correlatively to some chemotherapy drug sensitivity. Among them, Fluorouracil, Doxorubicin, lenvatinib and epirubicin can be used in the clinical treatment of liver cancer, and Lenvatinib is a first-line targeted drug for the treatment of HCC. The more specific details are presented in **Table S2** and **Table S5**. Those findings indicated that the model could act as a potential predictor for multidrug resistance-related genes and chemosensitivity.

### Validation of the differential expression of prognostic genes between HCC and adjacent non-tumorous tissues

**Figure 13:**
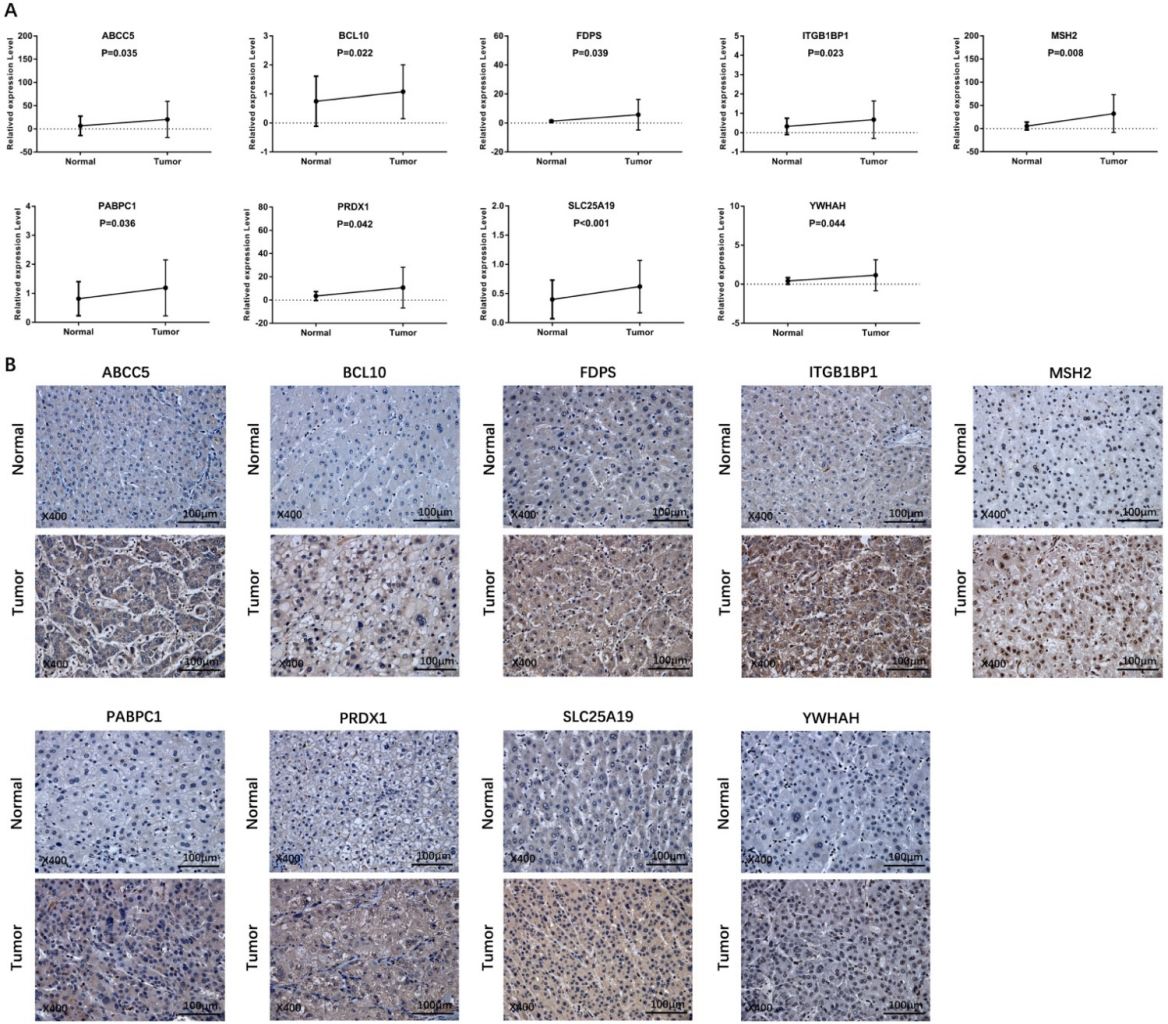
** Verification of the expression level of prognostic genes between HCC and adjacent normal tissue in an independent HCC cohort. (A)** The mRNA expression level of prognostic genes in HCC and adjacent normal tissue detected by qRT-PCR. **(B)** Representative IHC images of prognostic genes in tumor and adjacent normal tissue.

**Figure 14:**
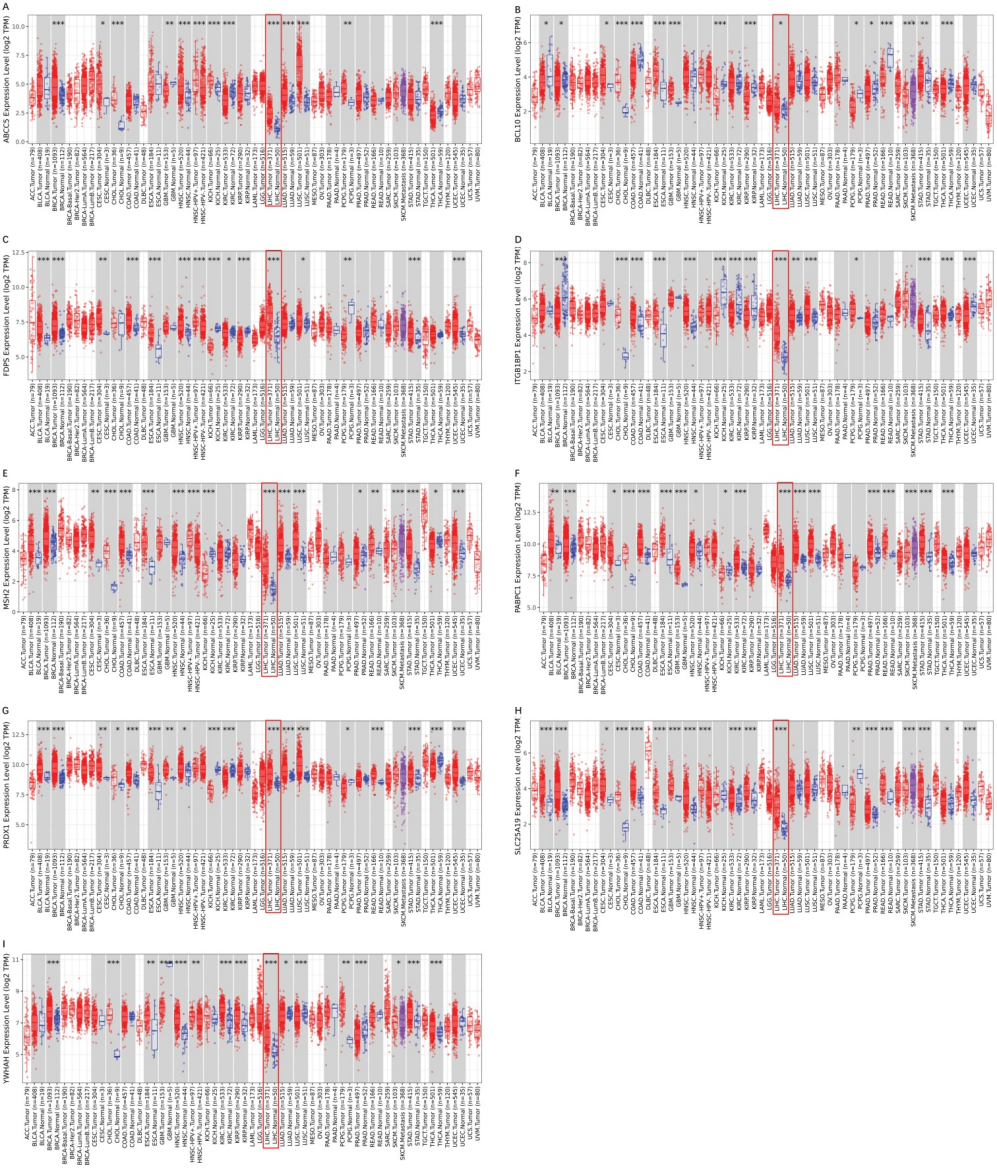
** Expression box diagram of gene expression in pan-cancer. (A-I)** The gene expression of *ABCC5, BCL10, FDPS, ITGB1BP1, MSH2, PABPC1, PRDX1, SLC25A19* and *YWHAH* between tumor and adjacent normal tissue in pan-cancer.

To validate the different mRNA and protein expression levels of the 9 prognostic genes (*ABCC5, BCL10, FDPS, ITGB1BP1, MSH2, PABPC1, PRDX1, SLC25A19,* and* YWHAH*) between HCC and adjacent non-tumorous tissues, qRT-PCR and IHC were performed. As shown in **Figure 13,** all the prognostic genes were highly expressed in HCC tissues than that in adjacent non-tumorous tissues, suggesting the same conclusion shown in the TCGA cohort and **Figure S3**. Moreover, we conducted comparisons of expression of prognostic genes between normal and tumor samples across TCGA cancer types in **Figure 14**. Compared with the expression levels in normal samples, *ABCC5, FDPS, ITGB1BP1, MSH2, PABPC1, PRDX1, SLC25A19* and* YWHAH* showed significantly high expression levels in most cancer types, while *BCL10* was expressed lowly in most cancer types except for CHOL, LIHC, ESCA and STAD. These findings indicated that the expression of most prognostic genes in other tumors was consistent with that of HCC, which could further study the application value of this model in other tumors. At the same time, it also showed a significant inter-tumor heterogeneity considering the expression levels of some prognostic genes.

## Discussion

HCC is a fatal disease with high incidence and poor prognosis 22, 23. A large number of HCC patients are diagnosed at an advanced stage, which means that effective treatments are mostly lost 24. The development of high-throughput sequencing provides an opportunity to identify biomarkers that predict prognosis for HCC and to enhance treatment to improve clinical outcomes for HCC 25, 26. Therefore, it is necessary to identify key molecular markers that can affect the prognosis of HCC, so as to better achieve individualized survival prediction with better accuracy.

In the present study, gene expression data were retrieved from TCGA and ICGC databases to classify prognostic DEGs between HCC and adjacent non-tumorous tissues. Then, a prognostic risk model including nine genes, which were selected by univariate Cox and LASSO Cox regression, was established. The risk model consisted of *ABCC5, BCL10, FDPS, ITGB1BP1, MSH2, PABPC1, PRDX1, SLC25A19,* and *YWHAH* was effective and stable to predict the prognosis of HCC patients through external validations.

Among the 9 prognostic genes, *ABCC5* is aberrantly upregulated in several human malignancies 27-29 and is responsible for the multidrug resistance phenotype causing HCC treatment failure based on the drug efflux pumps 30-32. *BCL10*, a key participator in the regulation of DNA double-strand breaks repair 33, is commonly involved in promoting the growth and invasion of cancer cells 34-36. Overexpression of *FDPS* leads to activation of oncogenic signaling and changes in the prenylation of small GTPases 37. ITGB1 functions as an oncogene in different types of human cancers 38, 39 and the overexpression of ITGB1 is found to promote the growth and metastasis of HCC cells 40. As far as we know, there is no report about *ITGB1BP* and HCC. The team of Hinrichsen pointed that up-regulation of* MSH2* is positively related to the occurrence and metastasis of HCC 41. Previous research strongly showed that *PABPC1* plays a role in HCC and can accelerate cell proliferation 42. High expression of *PRDX1* in HCC tissues corresponds to adverse clinical outcomes, and the mechanism may be related to promoting tumor angiogenesis and regulating cell migration and invasion 43-45. A recent report showed that *SLC25A19* is up-regulated in 43 breast cancer specimens, indicating its roles in breast cancer 46. The members of *YWHAH* are reported to be overexpressed in the cancerous area of various malignancies and they contribute to the carcinogenesis, including HCC 47-49. Given the importance of the 9 peroxisome-related genes in kinds of cancer types, these genes might be potential prognostic biomarkers for HCC patients. However, the specific molecular mechanism of the 9 genes in HCC needs further exploration.

Owing to the development of microarray and next-generation sequencing technologies, many multigene prognostic models have been developed to predict survival for HCC patients 50, 51. However, this is the first study about a prognostic model in HCC patients constructed using multiple peroxisome-related genes. The novel prognostic model was further evaluated under various clinical settings including survival. Based on the risk score signature, survival analysis displayed a significant difference in OS between high- and low-risk groups, and high-risk patients always encountered death earlier than low-risk patients. The ROC curves suggested that our prognostic model had good accuracy, and the AUC values of 1-, 2-, and 3-year showed the good predictive value of the model in both short- and long-term follow-ups. The findings above indicated that the prognostic model we constructed was capable of general application. Besides, the risk score was an independent prognostic indicator in HCC in both training set and testing set and the ability of prognostic prediction was further enhanced when the risk score combined with the tumor stage.

Furthermore, the immune status was different between the two groups. It is noteworthy that the high-risk group had a higher infiltration proportion of aDCs, iDCs, macrophages, and Th2 cells, as well as higher expression levels of immune checkpoints (PD-L2 and Galectin9). On the contrary, the patients in the high-risk group had lower activity of type II IFN response, which is described as a key to activate cell-mediated immune responses to control intracellular pathogens 52, suggesting the low anti-tumor immune response in the high-risk group. In terms of these immune cells, immune checkpoints, and multidrug resistance-related genes, the function of tumor infiltration has also been reported to be related to survival in HCC 53. ANOVA showed that the more aggressive subtypes of immune infiltration (C1 and C2) were observed in the high-risk group, suggesting a positive correlation of prognostic gene expression with poor prognosis. Collectively, the evidence for the association between risk scores and immune highlighted the importance of the peroxisome-related gene signature in prognostic prediction and treatment for HCC patients. Compared with other prognostic models of HCC, our analysis provides clear additional evidence that the risk score model based on the prognostic genes is directly linked to immune infiltration, immune pathways and immune checkpoints. However, further research, including *in vivo* and *in vitro* validation, as well as clinical trials, is needed to evaluate the correlation between peroxisome-related genes and immune regulation more accurately in HCC.

Cancer stem cells (CSCs) have been classified as a small subset of tumor cells with the characteristics to influence self-renewal and differentiation, which makes it hard to eliminate the tumor 54. CSCs have been identified in numerous solid tumors, such as breast cancer, colon cancer, and HCC 55-57. We further analyzed the relationship of the expression of peroxisome-related genes with RNAss in HCC. Interestingly, the findings were consistent with the fact that the expression of stem cells in various tumors correlates inversely with the outcome 58. This strongly suggested that the model we established had the ability to identify the score of CSCs and provided the possibility that targeting those genes might inhibit the process of HCC via affecting CSCs.

Accumulating evidence has indicated that chemotherapy of HCC is facing drug resistance, which leads to unsatisfactory therapeutic effect 59. The high expression of multidrug resistance-related genes and the decrease of drug sensitivity have drawn the eyes 60, 61. Thus, the further focus was on the prognostic model in the HCC chemotherapy. Consistently, the findings exhibited that MRP1, MRP3 and MRP5 were overexpressed in the high-risk group than that in low-risk group, and the lower drug sensitivity was observed in the higher expression levels of each prognostic gene. The results suggested that lower expression of prognostic genes benefited the treatment with chemotherapy of HCC patients.

## Conclusion

This study demonstrated that a novel signature constructed by the 9 peroxisome-related genes to predict the prognosis for HCC patients by the TCGA and ICGC cohorts. And we evaluated the novel prognostic model under various clinical settings including survival and clinical-pathological characteristics, immune infiltration, immune pathways, immune checkpoints, multidrug resistance-related genes and chemotherapy. Last, an independent sample cohort was carried out to validate the mRNA and protein expression levels of the 9 peroxisome-related genes. To sum up, this prognostic model can accurately predict the OS of HCC, distinguish the immune status of HCC, and the risk score is related to anti-tumor drugs. It is essential to systematically explore the potential role of peroxisome-related prognostic genes in HCC progression, and provided new possibilities for HCC therapeutic intervention.

## Supplementary Material

Supplementary figures and tables.

## Figures and Tables

**Table 1:** Clinical characteristics of the HCC patients used in this study

	TCGA-LIHC cohort	ICGC-LIRP-JI cohort
No. of patients	365	231
Age (median, range)	57 (16-90)	67 (31-89)
**Gender**		
Female	119 (32.6%)	61 (26.4%)
Male	246 (67.4%)	170 (73.6%)
**Grade**		
Grade 1	55 (15.1%)	NA
Grade 2	175 (47.9%)	NA
Grade 3	118 (32.3%)	NA
Grade 4	12 (3.3%)	NA
Unknown	5 (1.4%)	NA
**Stage**		
I	170 (46.6%)	36 (15.6%)
II	84 (23.0%)	105 (45.5%)
III	83 (22.7%)	71 (30.7%)
IV	4 (1.1%)	19 (8.2%)
Unknown	24 (6.6%)	0 (0%)
**Survival status**		
Alive	235 (64.4%)	189 (81.8%)
Deceased	130 (35.6%)	42 (18.2%)

**Table 2:** Baseline characteristics of the patients in different risk groups

Characteristics	TCGA-LIHC cohort			ICGC-LIRP-JI cohort		
	High risk	Low risk	P value	High risk	Low risk	P value
**Age**						
< 60 year	84(23.0%)	81(22.2%)	0.717	22(9.5%)	22(9.5%)	0.975
≥ 60 year	98(26.8)	102(27.9%)		93(40.3%)	94(40.7%)	
**Gender**						
Female	55(15.1%)	64(17.5%)	0.333	33(14.3%)	28(12.1%)	0.432
Male	127(34.8%)	119(32.6%)		82(35.5%)	88(38.1%)	
**Grade**						
G1+G2	97(26.6%)	133(36.4%)	<0.001	-	-	
G3+G4	83(22.7%)	47(12.9%)		-	-	
unknown	2(0.5%)	3(0.8%)		-	-	
**Stage**						
I + II	120(32.9%)	134(36.7%)	0.044	62(26.8%)	79(34.2%)	0.027
III + IV	52(14.2%)	35(9.6%)		53(22.9%)	37(16.0%)	
unknown	10(2.7%)	14(3.8%)		0(0.0%)	0(0.0%)	

## Abbreviations

HCC: hepatocellular carcinoma
TCGA: The Cancer Genome Atlas
ICGC: International Cancer Genome Consortium
OS: overall survival
DEGs: differentially expressed genes
LASSO: the least absolute shrinkage and selection operator
AUC: area under the curve
RNAss: stemness score based on mRNA expression
GO: Gene Ontology
KEGG: Kyoto Encyclopedia of Genes and Genomes
GSEA: gene set enrichment analysis
qRT-PCR: Real-time Quantitative-Polymerase Chain Reaction
ROC: receiver operating characteristic
IHC: immunohistochemistry

## References

[1] Forner A, Reig M, Bruix J (2018). Hepatocellular carcinoma. The Lancet.

[2] Wong MCS HJ, George J, Huang J, Leung C, Eslam M, Chan HLY, Ng SC (2019). The changing epidemiology of liver diseases in the Asia-Pacific region. Nat Rev Gastroenterol Hepatol.

[3] S M (2016). Involvement of ion channels and transporters in carcinoma angiogenesis and metastasis. Am J Physiol Cell Physiol. Am J Physiol Cell Physiol.

[4] Novikova MV, Khromova NV, Kopnin PB (2017). Components of the Hepatocellular Carcinoma Microenvironment and Their Role in Tumor Progression. Biochemistry (Mosc).

[5] Kulik L, Heimbach JK, Zaiem F, Almasri J, Prokop LJ, Wang Z (2018). Therapies for patients with hepatocellular carcinoma awaiting liver transplantation: A systematic review and meta-analysis. Hepatology.

[6] Wang J, Ha J, Lopez A, Bhuket T, Liu B, Wong RJ (2018). Medicaid and Uninsured Hepatocellular Carcinoma Patients Have More Advanced Tumor Stage and Are Less Likely to Receive Treatment. Journal of Clinical Gastroenterology.

[7] Nakagawa H, Fujita M, Fujimoto A (2019). Genome sequencing analysis of liver cancer for precision medicine. Semin Cancer Biol.

[8] Zucman-Rossi J, Villanueva A, Nault JC, Llovet JM (2015). Genetic Landscape and Biomarkers of Hepatocellular Carcinoma. Gastroenterology.

[9] Zeng XC, Zhang L, Liao WJ, Ao L, Lin ZM, Kang W (2020). Screening and Identification of Potential Biomarkers in Hepatitis B Virus-Related Hepatocellular Carcinoma by Bioinformatics Analysis. Front Genet.

[10] Dickinson BC, Chang CJ (2011). Chemistry and biology of reactive oxygen species in signaling or stress responses. Nat Chem Biol.

[11] Reczek CR, Chandel NS (2015). ROS-dependent signal transduction. Curr Opin Cell Biol.

[12] Rabinovitch RC, Samborska B, Faubert B, Ma EH, Gravel SP, Andrzejewski S (2017). AMPK Maintains Cellular Metabolic Homeostasis through Regulation of Mitochondrial Reactive Oxygen Species. Cell Rep.

[13] Pljesa-Ercegovac M, Mimic-Oka J, Dragicevic D, Savic-Radojevic A, Opacic M, Pljesa S (2008). Altered antioxidant capacity in human renal cell carcinoma: role of glutathione associated enzymes. Urol Oncol.

[14] Frederiks WM BK, Hoeben KA, van Marle J, Langbein S (2010). Renal cell carcinoma and oxidative stress: The lack of peroxisomes. Acta Histochem.

[15] Dhaunsi GS GS, Singh AK, Orak JK, Asayama K, Singh I (1992). Demonstration of Cu-Zn superoxide dismutase in rat liver peroxisomes. Biochemical and immunochemical evidence. Biol Chem.

[16] Zhang X, Yang H, Zhang J, Gao F, Dai L (2020). HSD17B4, ACAA1, and PXMP4 in Peroxisome Pathway Are Down-Regulated and Have Clinical Significance in Non-small Cell Lung Cancer. Front Genet.

[17] Thorsson V GD, Brown SD, Wolf D, Bortone DS, Ou Yang TH, Porta-Pardo E (2018). The Immune Landscape of Cancer. Immunity.

[18] Malta TM SA, Gentles AJ, Burzykowski T, Poisson L, Weinstein JN, Kamińska B (2018). Machine Learning Identifies Stemness Features Associated with Oncogenic Dedifferentiation. Cell.

[19] Tamborero D, Rubio-Perez C, Muinos F, Sabarinathan R, Piulats JM, Muntasell A (2018). A Pan-cancer Landscape of Interactions between Solid Tumors and Infiltrating Immune Cell Populations. Clin Cancer Res.

[20] Du J YX, Mi S, Li Y, Ji H, Hou K, Ma S (2020). Identification of Prognostic Model and Biomarkers for Cancer Stem Cell Characteristics in Glioblastoma by Network Analysis of Multi-Omics Data and Stemness Indices. Front Cell Dev Biol.

[21] Zhang Y TB, Song J, Yu S, Li Y, Su H, He S (2019). Lnc-PDZD7 contributes to stemness properties and chemosensitivity in hepatocellular carcinoma through EZH2-mediated ATOH8 transcriptional repression. Exp Clin Cancer Res.

[22] Planchard D, Smit EF, Groen HJM, Mazieres J, Besse B, Helland Å (2017). Dabrafenib plus trametinib in patients with previously untreated BRAFV600E-mutant metastatic non-small-cell lung cancer: an open-label, phase 2 trial. The Lancet Oncology.

[23] Roviello G, D'Angelo A, Petrioli R, Roviello F, Cianchi F, Nobili S (2020). Encorafenib, Binimetinib, and Cetuximab in BRAF V600E-Mutated Colorectal Cancer. Transl Oncol.

[24] Sun X, Ou Z, Chen R, Niu X, Chen D, Kang R (2016). Activation of the p62-Keap1-NRF2 pathway protects against ferroptosis in hepatocellular carcinoma cells. Hepatology.

[25] Zeng H, Zheng R, Guo Y, Zhang S, Zou X, Wang N (2015). Cancer survival in China, 2003-2005: a population-based study. Int J Cancer.

[26] Nakamura M, Chiba T, Kanayama K, Kanzaki H, Saito T, Kusakabe Y (2019). Epigenetic dysregulation in hepatocellular carcinoma: an up-to-date review. Hepatology Research.

[27] Borst P, de Wolf C, van de Wetering K (2007). Multidrug resistance-associated proteins 3, 4, and 5. Pflugers Arch.

[28] Dean M AR (2001). Complete characterization of the human ABC gene family. J Bioenerg Biomembr. Bioenerg Biomembr.

[29] Mourskaia AA AE, Dong Z, Tiedemann K, Cory S, Omeroglu A, Bertos N (2012). ABCC5 supports osteoclast formation and promotes breast cancer metastasis to bone. Breast Cancer Res Treat.

[30] Tian Q ZJ, Chan SY, Tan TM, Duan W, Huang M, Zhu YZ (2006). Topotecan is a substrate for multidrug resistance associated protein 4. Curr Drug Metab.

[31] Hopper-Borge E CZ, Shchaveleva I, Belinsky MG, Kruh GD (2004). Analysis of the drug resistance profile of multidrug resistance protein 7 (ABCC10): resistance to docetaxel. Cancer Research.

[32] Lagas JS VM, Schinkel AH (2009). Pharmacokinetic assessment of multiple ATP-binding cassette transporters: the power of combination knockout mice. Mol Interv.

[33] Ismail IH, Dronyk A, Hu X, Hendzel MJ, Shaw AR (2016). BCL10 is recruited to sites of DNA damage to facilitate DNA double-strand break repair. Cell Cycle.

[34] Shang Y LY, Zhang Y, Wang J (2019). ZNF436 promotes tumor cell proliferation through transcriptional activation of BCL10 in glioma. Biochem Biophys Res Commun.

[35] Phelan JD YR, Webster DE, Roulland S, Wright GW, Kasbekar M, Shaffer AL 3rd (2018). A multiprotein supercomplex controlling oncogenic signalling in lymphoma. Nature.

[36] Ekambaram P LJ, Hubel NE, Hu D, Yerneni S, Campbell PG, Pollock N (2018). The CARMA3-Bcl10-MALT1 Signalosome Drives NFκB Activation and Promotes Aggressiveness in Angiotensin II Receptor-Positive Breast Cancer. Cancer Res.

[37] Seshacharyulu P, Rachagani S, Muniyan S, Siddiqui JA, Cruz E, Sharma S (2019). FDPS cooperates with PTEN loss to promote prostate cancer progression through modulation of small GTPases/AKT axis. Oncogene.

[38] Kurozumi A GY, Matsushita R, Fukumoto I, Kato M, Nishikawa R, Sakamoto S (2016). Tumor-suppressive microRNA-223 inhibits cancer cell migration and invasion by targeting ITGA3/ITGB1 signaling in prostate cancer. Cancer Sci.

[39] Hunt S JA, Hinsley EE, Whawell SA, Lambert DW (2011). MicroRNA-124 suppresses oral squamous cell carcinoma motility by targeting ITGB1. FEBS Lett.

[40] Zhang YY KL, Zhu XD, Cai H, Wang CH, Shi WK, Cao MQ (2018). CD31 regulates metastasis by inducing epithelial-mesenchymal transition in hepatocellular carcinoma via the ITGB1-FAK-Akt signaling pathway. Cancer Lett.

[41] Hinrichsen I KM, Peveling-Oberhag J, Passmann S, Plotz G, Zeuzem S, Brieger A (2014). Promoter methylation of MLH1, PMS2, MSH2 and p16 is a phenomenon of advanced-stage HCCs. PLoS One.

[42] Huo X HS, Wu G, Latchoumanin O, Zhou G, Hebbard L, George J, Qiao L (2017). Dysregulated long noncoding RNAs (lncRNAs) in hepatocellular carcinoma: implications for tumorigenesis, disease progression, and liver cancer stem cells. Mol Cancer.

[43] Aguilar-Melero P, Prieto-Alamo MJ, Jurado J, Holmgren A, Pueyo C (2013). Proteomics in HepG2 hepatocarcinoma cells with stably silenced expression of PRDX1. J Proteomics.

[44] Deng L, Gan X, Ito M, Chen M, Aly HH, Matsui C (2019). Peroxiredoxin 1, a Novel HBx-Interacting Protein, Interacts with Exosome Component 5 and Negatively Regulates Hepatitis B Virus (HBV) Propagation through Degradation of HBV RNA. J Virol.

[45] Kang MA, Jeon YK, Nam MJ Auricularia auricula increases an apoptosis in human hepatocellular carcinoma cells via a regulation of the peroxiredoxin1. J Food Biochem. 2020: e13373.

[46] Zastre JA HB, Sweet RL, McGinnis AC, Venuti KR, Bartlett MG, Govindarajan R (2013). Up-regulation of vitamin B1 homeostasis genes in breast cancer. Nutr Biochem.

[47] Tseng CW YJ, Chen CN, Huang HC, Chuang KN, Lin CC, Lai HS (2011). Identification of 14-3-3β in human gastric cancer cells and its potency as a diagnostic and prognostic biomarker. Proteomics. Proteomics.

[48] Xiao Y LV, Ke S, Lin GE, Lin FT, Lin WC (2014). 14-3-3τ promotes breast cancer invasion and metastasis by inhibiting RhoGDIα. Mol Cell Biol.

[49] Iizuka N, Tsunedomi R, Tamesa T, Okada T, Sakamoto K, Hamaguchi T (2006). Involvement of c-myc-regulated genes in hepatocellular carcinoma related to genotype-C hepatitis B virus. J Cancer Res Clin Oncol.

[50] Man Z CY, Gao L, Xei G, Li Q, Lu Q, Yan J (2021). A Prognostic Model Based on RNA Binding Protein Predicts Clinical Outcomes in Hepatocellular Carcinoma Patients. Front Oncol.

[51] Dessie EY TS, Chiang HS, Tsai JJP, Chang YS, Chang JG, Ng KL (2021). Construction and Validation of a Prognostic Gene-Based Model for Overall Survival Prediction in Hepatocellular Carcinoma Using an Integrated Statistical and Bioinformatic Approach. Int J Mol Sci.

[52] Yap GS SA (1999). Cell-mediated immunity to Toxoplasma gondii: initiation, regulation and effector function. Immunobiology.

[53] Peng Y LC, Li M, Li W, Zhang M, Jiang X, Chang Y (2021). Identification of a prognostic and therapeutic immune signature associated with hepatocellular carcinoma. Cancer Cell Int.

[54] Liu YC YC, Lin KH (2020). Cancer Stem Cell Functions in Hepatocellular Carcinoma and Comprehensive Therapeutic Strategies. Cells.

[55] Rigiracciolo DC CF, Talia M, Muglia L, Gutkind JS, Maggiolini M, Lappano R (2021). Focal Adhesion Kinase Fine Tunes Multifaced Signals toward Breast Cancer Progression. Cancers (Basel).

[56] Stark VA FC, Viswanathan V, Boman BM (2021). The Role of miRNAs, miRNA Clusters, and isomiRs in Development of Cancer Stem Cell Populations in Colorectal Cancer. Int J Mol Sci.

[57] Dai X GY, Hu Y, Bao X, Zhu X, Fu Q, Zhang H (2021). Immunotherapy for targeting cancer stem cells in hepatocellular carcinoma. Theranostics.

[58] Merlos-Suárez A BF, Jung P, Iglesias M, Céspedes MV, Rossell D, Sevillano M (2011). The intestinal stem cell signature identifies colorectal cancer stem cells and predicts disease relapse. Cell Stem Cell.

[59] Liu XQ CX, Liu ZZ, Gu SS, He LJ, Wang KP, Tang RZ (2020). Biomimetic Matrix Stiffness Modulates Hepatocellular Carcinoma Malignant Phenotypes and Macrophage Polarization through Multiple Modes of Mechanical Feedbacks. ACS Biomater Sci Eng.

[60] Barraud L MP, Soma E, Lefrançois L, Guerret S, Chevallier M, Dubernet C (2005). Increase of doxorubicin sensitivity by doxorubicin-loading into nanoparticles for hepatocellular carcinoma cells *in vitro* and *in vivo*. Hepatology.

[61] Li Y TX, Liu X, Liu L, Fang Y, Rao R, Ren Y (2020). Enhanced anticancer effect of doxorubicin by TPGS-coated liposomes with Bcl-2 siRNA-corona for dual suppression of drug resistance. Asian J Pharm Sci.

