# Supplementary material for: Establishment and Validation of a Peroxisome-related Gene Signature for Prognostic Prediction and Immune Distinction in Hepatocellular Carcinoma
Source: J Cancer. 2022 Feb 28;13(5):1418–35. doi: 10.7150/jca.65080 (PMC8965136; doi:10.7150/jca.65080)
Supplement: Supplementary file 1 — Supplementary figures and tables. [file jcav13p1418s1.pdf]

Figure S1. Construction of a 9-gene signature model in the TCGA cohort

(A) The confidence interval under each lambda. (B) The changing trajectory of each independent variable. The horizontal axis represents the log value of the independent variable lambda, and the vertical axis represents the coefficient of the independent variable

Figure S2. Survival analysis of each prognostic gene according to the optimal cut-off expression value in the TGCA cohort

Figure S3. Expression of each prognostic gene between HCC and adjacent non-tumorous tissues in TCGA

Figure S4. Expression levels of each prognostic gene in the different clinical feature groups

(A) Age (B) Gender (C) Histological grade (D) Tumor stage

Figure S5. Expression levels of each prognostic gene in the different immune infiltrate subtypes

Figure S6. Correlation matrixes between the prognostic gene expression and RNAss

Figure S7. Functional Enrichment of GO in the high-risk group by GSEA analysis

GSEA, gene set enrichment analysis

Figure S8. Functional Enrichment of KEGG in the high-risk group by GSEA analysis

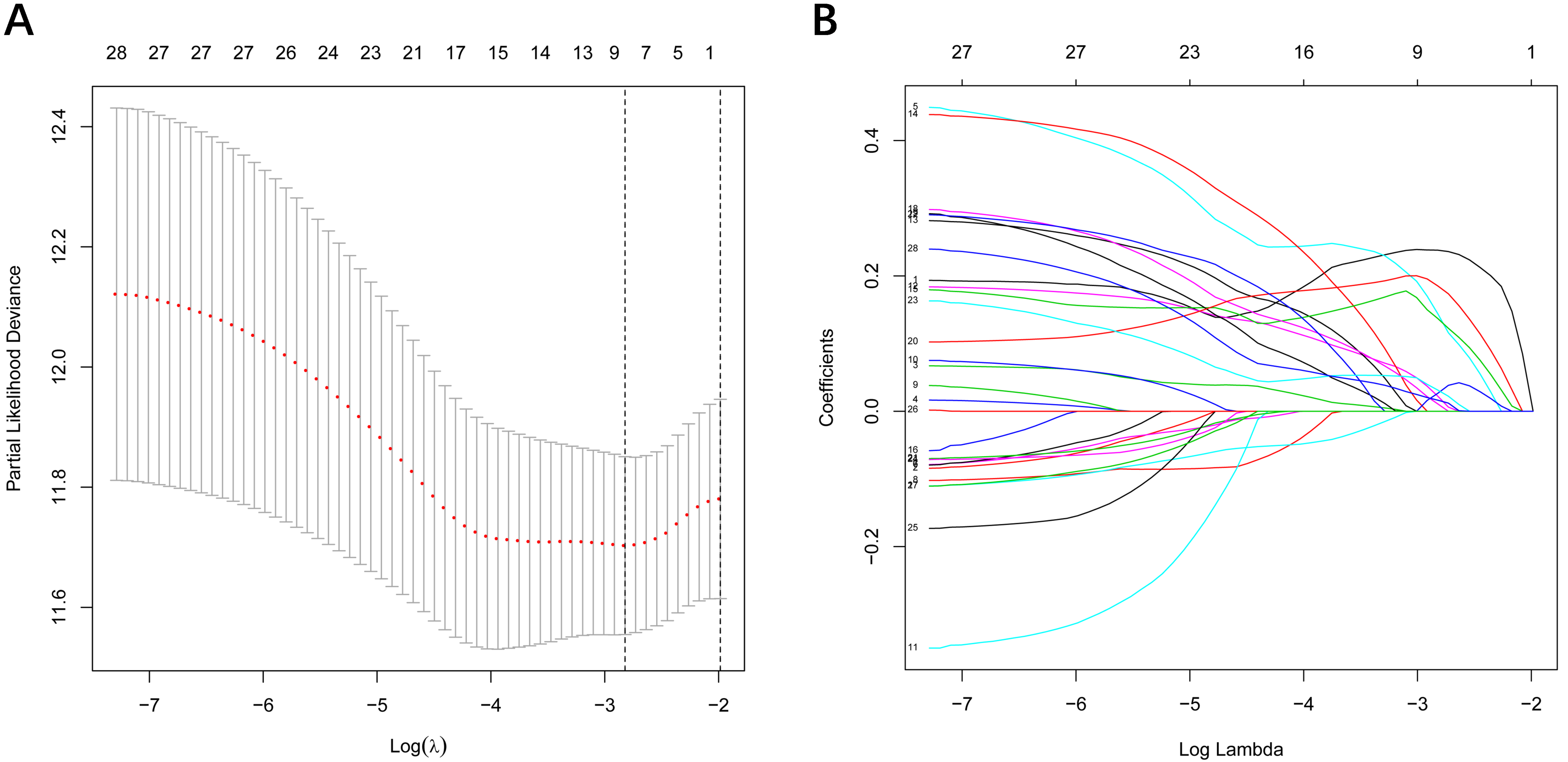
A
28 27 27 27 26 24 23 21 17 15 14 13 9 7 5 1
Partial Likelihood Deviance
12.4
12.2
12.0
11.8
11.6
-7 -6 -5 -4 -3 -2
Log(λ)
B
27 27 23 16 9 1
Coefficients
0.4
0.2
0.0
-0.2
-7 -6 -5 -4 -3 -2
Log Lambda

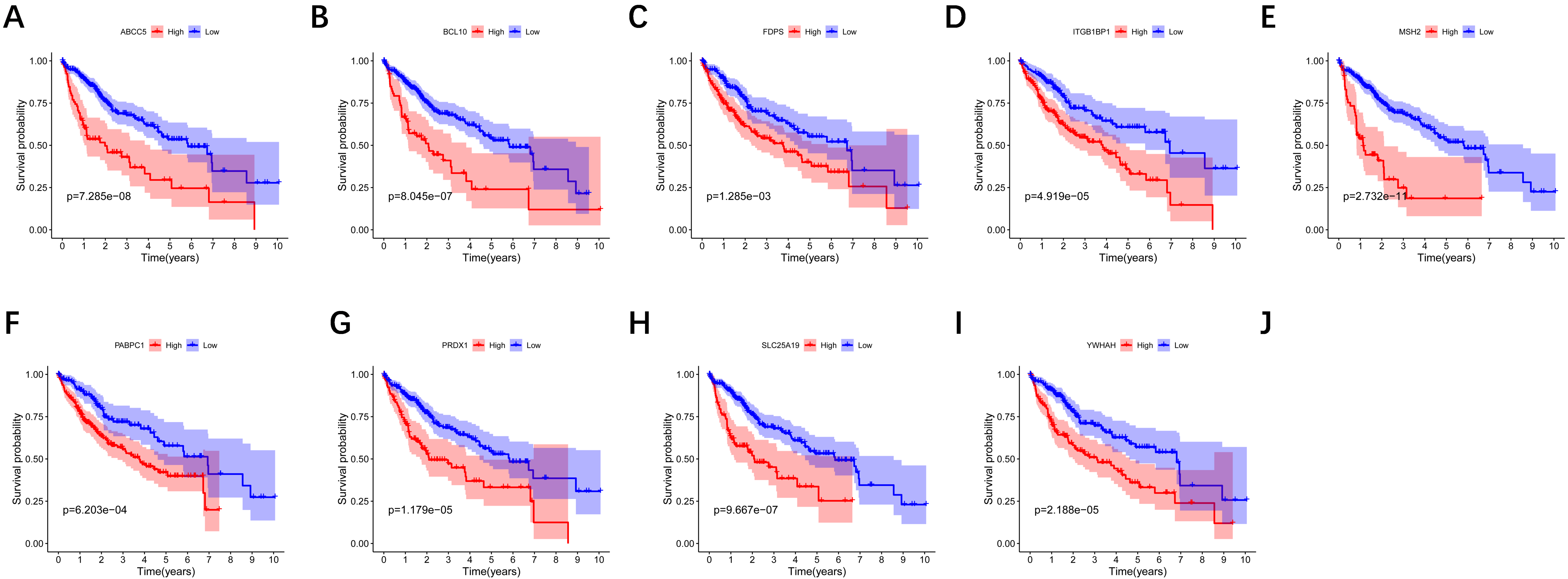
A
ABCC5 High Low
Survival probability
Time(years)
p=7.285e-08
B
BCL10 High Low
Survival probability
Time(years)
p=8.045e-07
C
FDPS High Low
Survival probability
Time(years)
p=1.285e-03
D
ITGB1BP1 High Low
Survival probability
Time(years)
p=4.919e-05
E
MSH2 High Low
Survival probability
Time(years)
p=2.732e-11
F
PABPC1 High Low
Survival probability
Time(years)
p=6.203e-04
G
PRDX1 High Low
Survival probability
Time(years)
p=1.179e-05
H
SLC25A19 High Low
Survival probability
Time(years)
p=9.667e-07
I
YWHAH High Low
Survival probability
Time(years)
p=2.188e-05
J

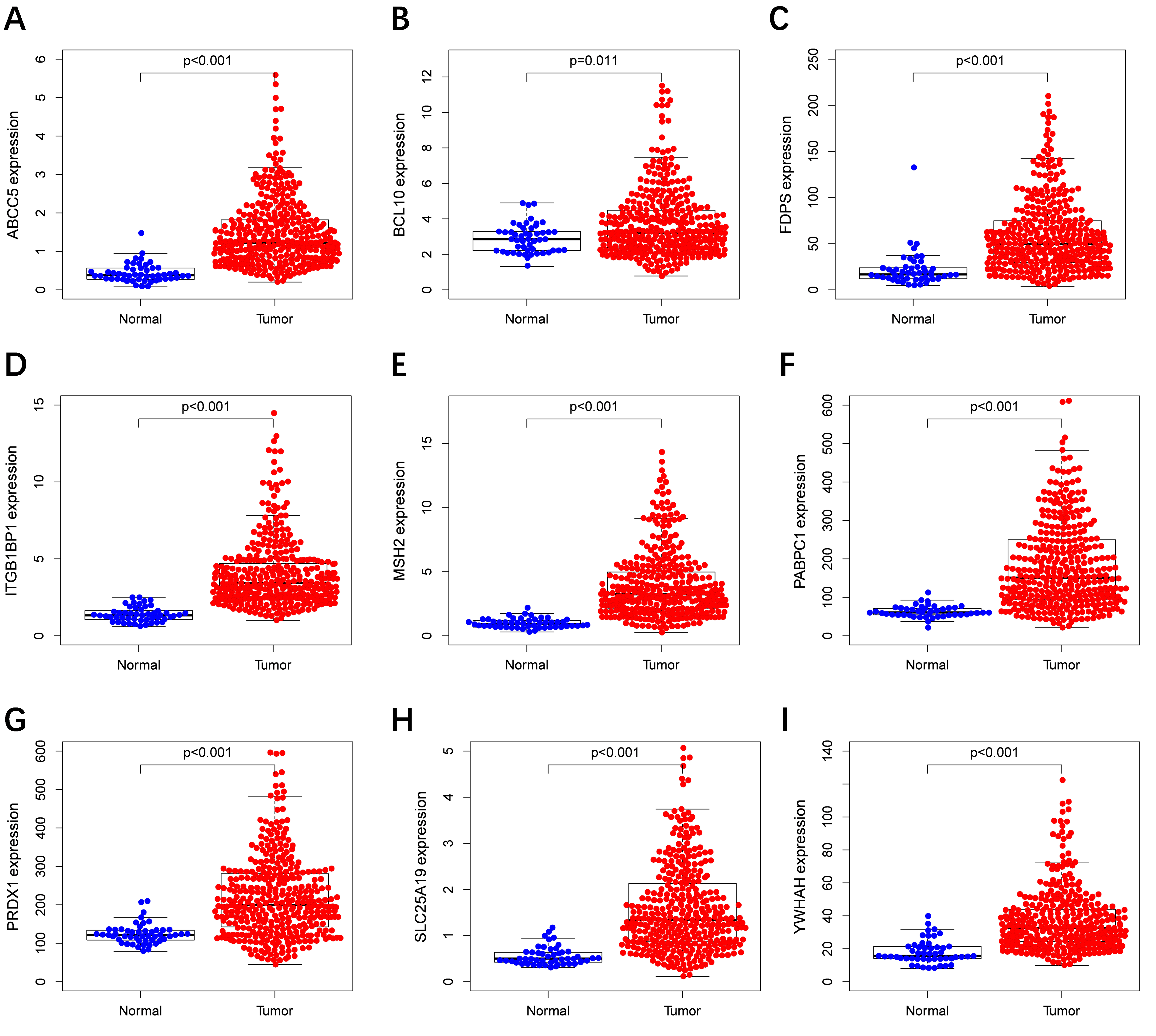
A
B
C
D
E
F
G
H
I
ABCC5 expression
BCL10 expression
FDPS expression
ITGB1BP1 expression
MSH2 expression
PABPC1 expression
PRDX1 expression
SLC25A19 expression
YWHAH expression
p<0.001
p=0.011
Normal
Tumor

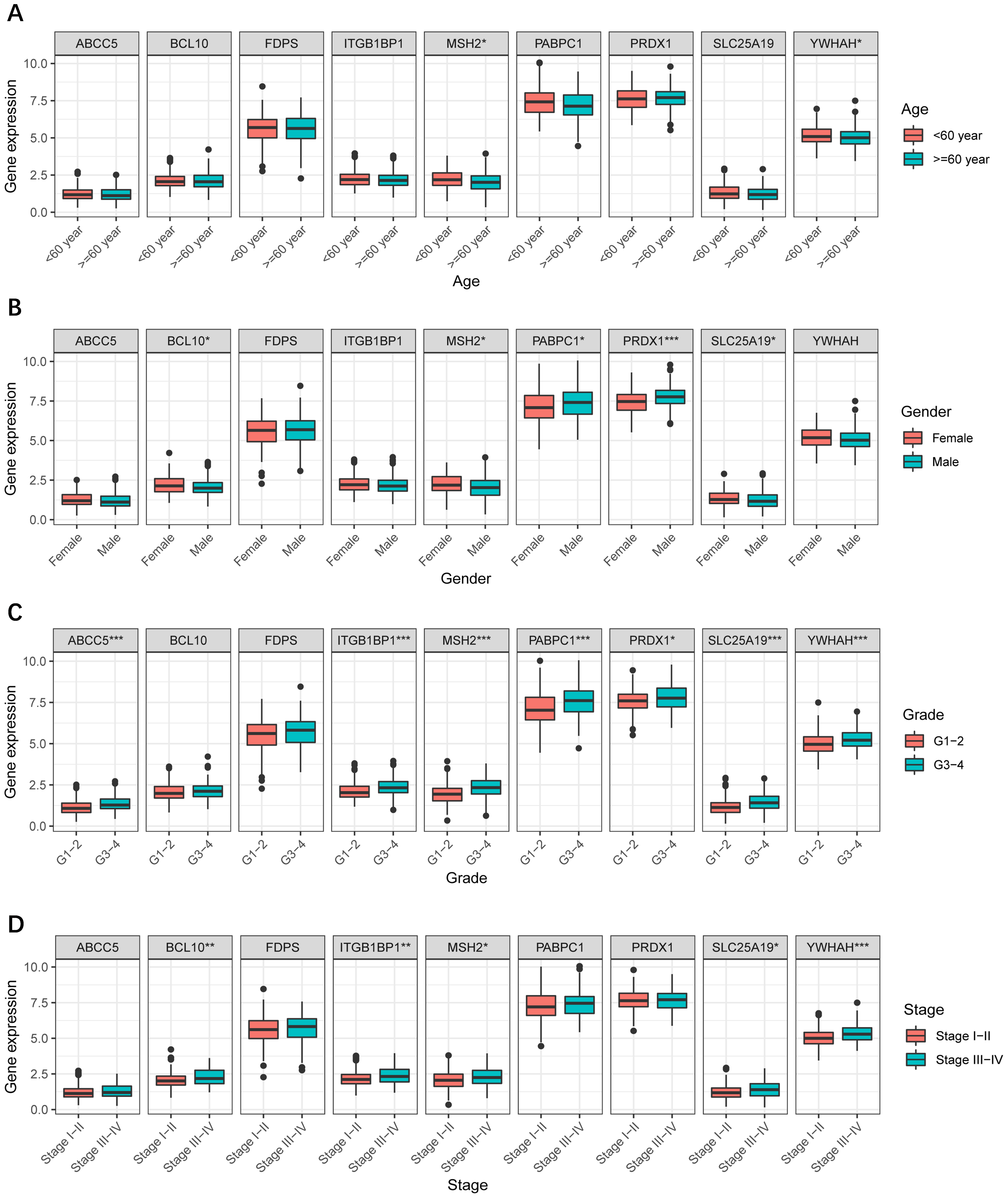
A
B
C
D
Gene expression
Age
Gender
Grade
Stage
<60 year
>=60 year
Female
Male
G1−2
G3−4
Stage I−II
Stage III−IV
ABCC5
BCL10
FDPS
ITGB1BP1
MSH2*
PABPC1
PRDX1
SLC25A19
YWHAH*
BCL10*
PABPC1*
PRDX1***
SLC25A19*
YWHAH
ABCC5***
ITGB1BP1***
MSH2***
PABPC1***
PRDX1*
SLC25A19***
YWHAH***
BCL10**
ITGB1BP1**

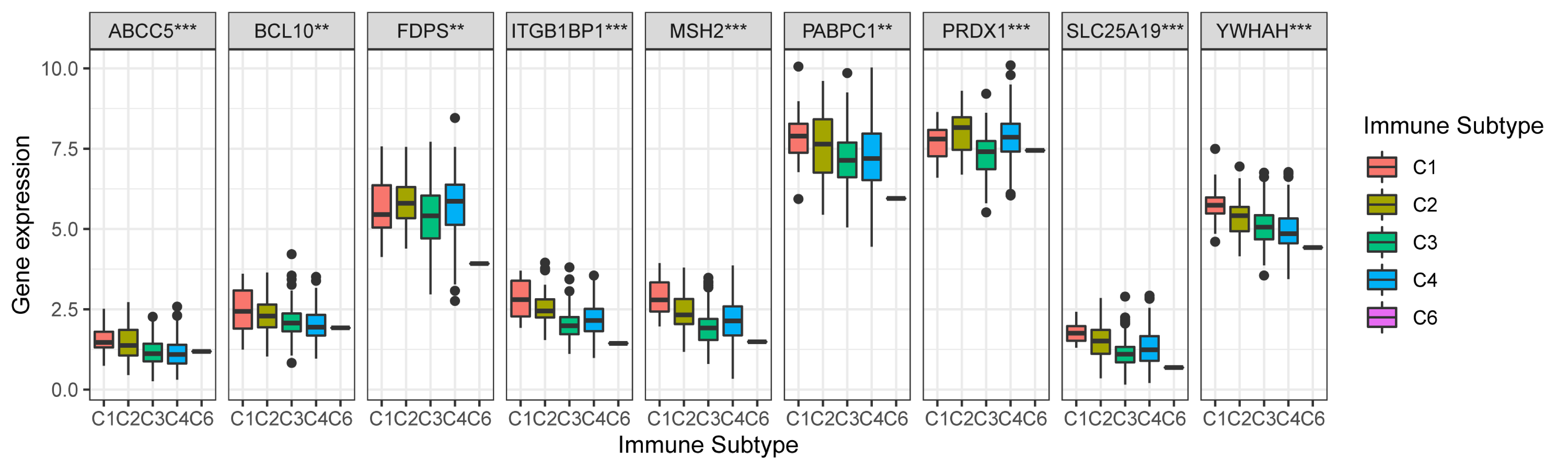

ABCC5***
BCL10**
FDPS**
ITGB1BP1***
MSH2***
PABPC1**
PRDX1***
SLC25A19***
YWHAH***
10.0
7.5
5.0
2.5
0.0
Gene expression
C1C2C3C4C6
Immune Subtype
Immune Subtype
C1
C2
C3
C4
C6

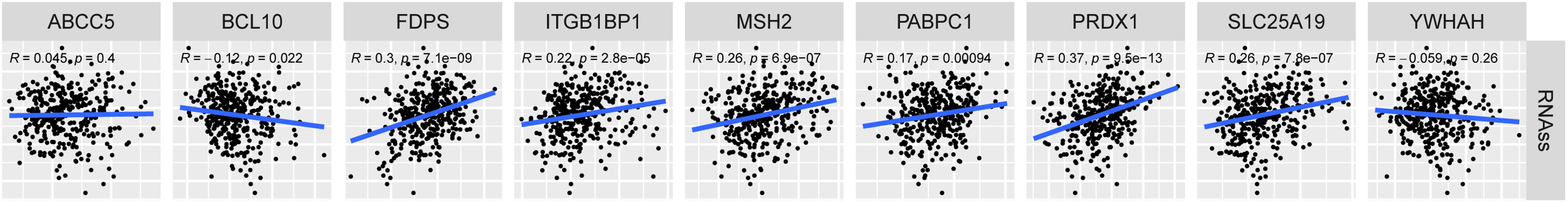
ABCC5
R = 0.045, p = 0.4
BCL10
R = −0.12, p = 0.022
FDPS
R = 0.3, p = 7.1e−09
ITGB1BP1
R = 0.22, p = 2.8e−05
MSH2
R = 0.26, p = 6.9e−07
PABPC1
R = 0.17, p = 0.00094
PRDX1
R = 0.37, p = 9.5e−13
SLC25A19
R = 0.26, p = 7.8e−07
YWHAH
R = −0.059, p = 0.26
RNAss

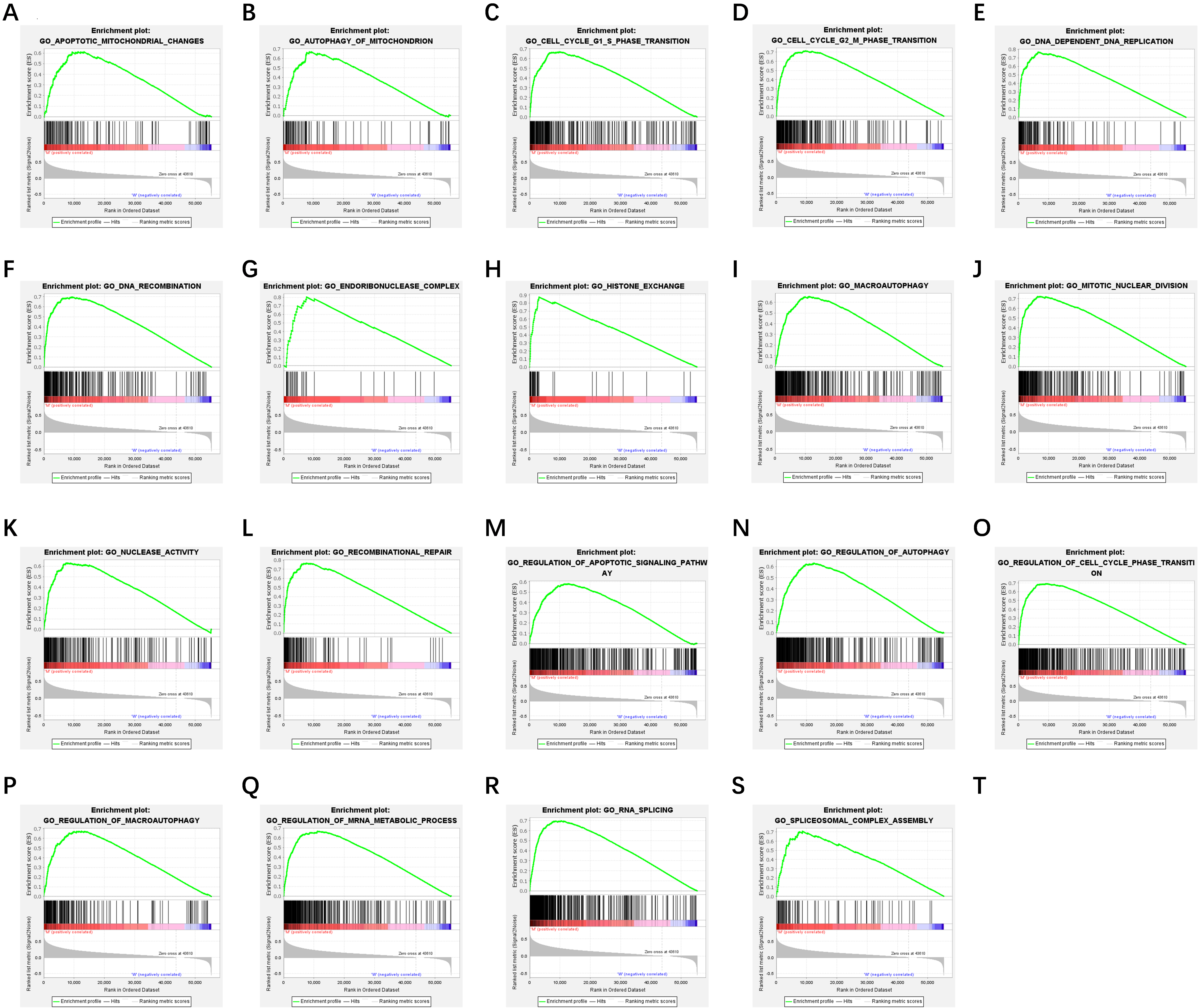
A
Enrichment plot: GO_APOPTOTIC_MITOCHONDRIAL_CHANGES
B
Enrichment plot: GO_AUTOPHAGY_OF_MITOCHONDRION
C
Enrichment plot: GO_CELL_CYCLE_G1_S_PHASE_TRANSITION
D
Enrichment plot: GO_CELL_CYCLE_G2_M_PHASE_TRANSITION
E
Enrichment plot: GO_DNA_DEPENDENT_DNA_REPLICATION
F
Enrichment plot: GO_DNA_RECOMBINATION
G
Enrichment plot: GO_ENDORIBONUCLEASE_COMPLEX
H
Enrichment plot: GO_HISTONE_EXCHANGE
I
Enrichment plot: GO_MACROAUTOPHAGY
J
Enrichment plot: GO_MITOTIC_NUCLEAR_DIVISION
K
Enrichment plot: GO_NUCLEASE_ACTIVITY
L
Enrichment plot: GO_RECOMBINATIONAL_REPAIR
M
Enrichment plot: GO_REGULATION_OF_APOPTOTIC_SIGNALING_PATHWAY
N
Enrichment plot: GO_REGULATION_OF_AUTOPHAGY
O
Enrichment plot: GO_REGULATION_OF_CELL_CYCLE_PHASE_TRANSITION
P
Enrichment plot: GO_REGULATION_OF_MACROAUTOPHAGY
Q
Enrichment plot: GO_REGULATION_OF_MRNA_METABOLIC_PROCESS
R
Enrichment plot: GO_RNA_SPLICING
S
Enrichment plot: GO_SPLICEOSOMAL_COMPLEX_ASSEMBLY
T
Enrichment score (ES)
Ranked list metric (Signal2Noise)
'M' (positively correlated)
'W' (negatively correlated)
Zero cross at 43610
Rank in Ordered Dataset
Enrichment profile
Hits
Ranking metric scores

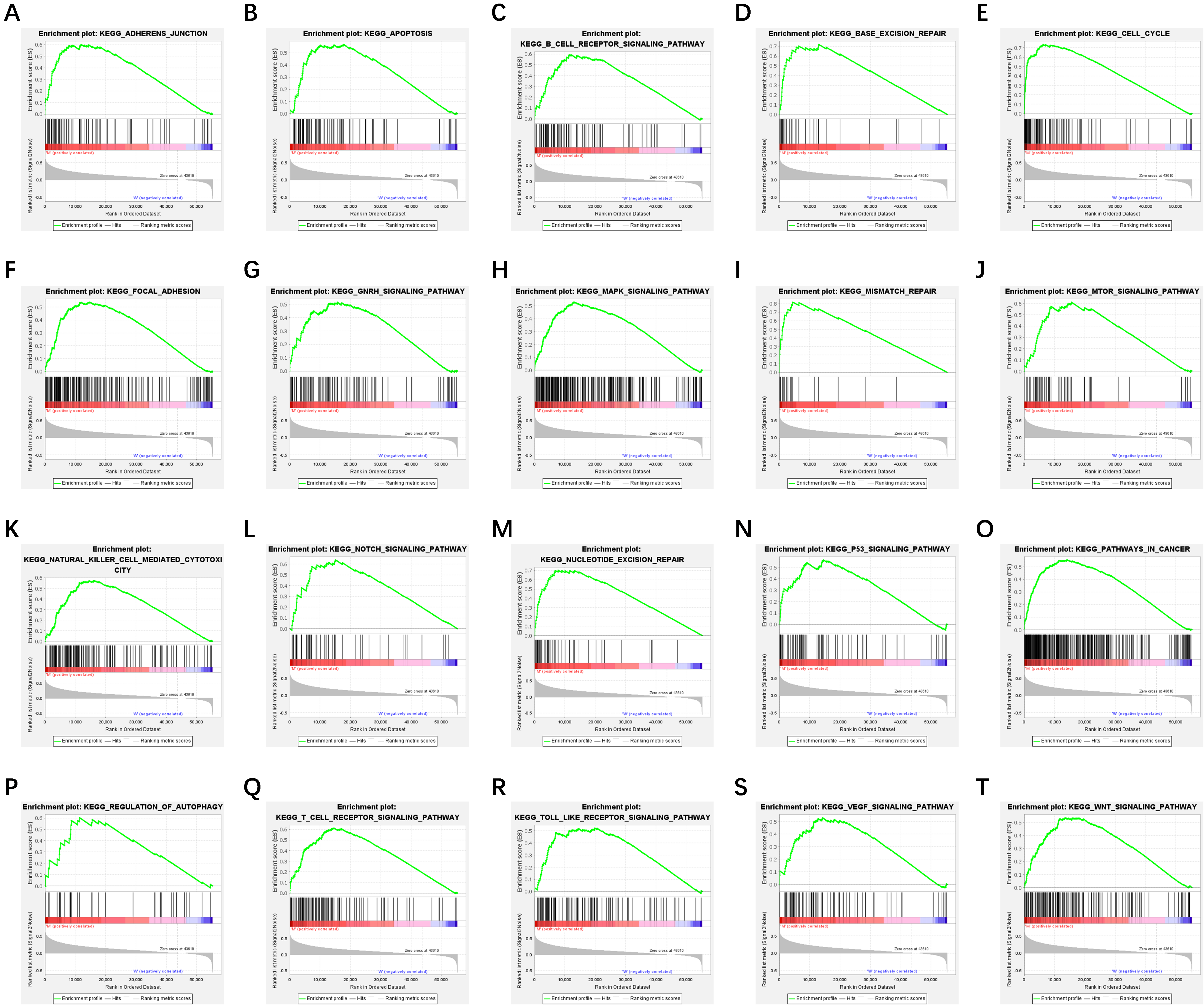
A
Enrichment plot: KEGG_ADHERENS_JUNCTION
B
Enrichment plot: KEGG_APOPTOSIS
C
Enrichment plot: KEGG_B_CELL_RECEPTOR_SIGNALING_PATHWAY
D
Enrichment plot: KEGG_BASE_EXCISION_REPAIR
E
Enrichment plot: KEGG_CELL_CYCLE
F
Enrichment plot: KEGG_FOCAL_ADHESION
G
Enrichment plot: KEGG_GNRH_SIGNALING_PATHWAY
H
Enrichment plot: KEGG_MAPK_SIGNALING_PATHWAY
I
Enrichment plot: KEGG_MISMATCH_REPAIR
J
Enrichment plot: KEGG_MTOR_SIGNALING_PATHWAY
K
Enrichment plot: KEGG_NATURAL_KILLER_CELL_MEDIATED_CYTOTOXICITY
L
Enrichment plot: KEGG_NOTCH_SIGNALING_PATHWAY
M
Enrichment plot: KEGG_NUCLEOTIDE_EXCISION_REPAIR
N
Enrichment plot: KEGG_P53_SIGNALING_PATHWAY
O
Enrichment plot: KEGG_PATHWAYS_IN_CANCER
P
Enrichment plot: KEGG_REGULATION_OF_AUTOPHAGY
Q
Enrichment plot: KEGG_T_CELL_RECEPTOR_SIGNALING_PATHWAY
R
Enrichment plot: KEGG_TOLL_LIKE_RECEPTOR_SIGNALING_PATHWAY
S
Enrichment plot: KEGG_VEGF_SIGNALING_PATHWAY
T
Enrichment plot: KEGG_WNT_SIGNALING_PATHWAY
Enrichment score (ES)
Ranked list metric (Signal2Noise)
'M' (positively correlated)
'W' (negatively correlated)
Zero cross at 43610
Rank in Ordered Dataset
Enrichment profile — Hits Ranking metric scores

Supplementary Table 1. The detailed 104 peroxisome-related genes

Gene Symbol	Gene Description	Gene Symbol	Gene Description
ABCB1	ATP binding cassette subfamily B member 1	EPHX2	epoxide hydrolase 2
ABCB4	ATP binding cassette subfamily B member 4	ERCC1	ERCC excision repair 1, endonuclease non-ca[illegible] subunit
ABCB9	ATP binding cassette subfamily B member 9	ERCC3	ERCC excision repair 3, TFIIH core complex h[illegible] subunit
ABCC5	ATP binding cassette subfamily C member 5	ESR2	estrogen receptor 2
ABCC8	ATP binding cassette subfamily C member 8	FABP6	fatty acid binding protein 6
ABCD1	ATP binding cassette subfamily D member 1	FADS1	fatty acid desaturase 1
ABCD2	ATP binding cassette subfamily D member 2	FDPS	farnesyl diphosphate synthase
ABCD3	ATP binding cassette subfamily D member 3	FIS1	fission, mitochondrial 1
ACAA1	acetyl-CoA acyltransferase 1	GNPAT	glyceronephosphate O-acyltransferase
ACOT8	acyl-CoA thioesterase 8	GSTK1	glutathione S-transferase kappa 1
ACOX1	acyl-CoA oxidase 1	HAO2	hydroxyacid oxidase 2
ACSL1	acyl-CoA synthetase long chain family member 1	HMGCL	3-hydroxy-3-methylglutaryl-CoA lyase
ACSL4	acyl-CoA synthetase long chain family member 4	HRAS	HRas proto-oncogene, GTPase
ACSL5	acyl-CoA synthetase long chain family member 5	HSD11B2	hydroxysteroid 11-beta dehydrogenase 2
ALB	albumin	HSD17B11	hydroxysteroid 17-beta dehydrogenase 11
ALDH1A1	aldehyde dehydrogenase 1 family member A1	HSD17B4	hydroxysteroid 17-beta dehydrogenase 4
ALDH9A1	aldehyde dehydrogenase 9 family member A1	HSD3B7	hydroxy-delta-5-steroid dehydrogenase, 3 bet[illegible] steroid delta-isomerase 7
ATXN1	ataxin 1	IDE	insulin degrading enzyme
BCL10	BCL10 immune signaling adaptor	IDH1	isocitrate dehydrogenase (NADP(+)) 1
CACNA1B	calcium voltage-gated channel subunit alpha1 B	IDH2	isocitrate dehydrogenase (NADP(+)) 2
CADM1	cell adhesion molecule 1	IDI1	isopentenyl-diphosphate delta isomerase 1
CAT	catalase	ISOC1	isochorismatase domain containing 1
CDK7	cyclin dependent kinase 7	ITGB1BP1	integrin subunit beta 1 binding protein 1
CEL	carboxyl ester lipase	LONP2	lon peptidase 2, peroxisomal
CLN6	CLN6 transmembrane ER protein	MLYCD	malonyl-CoA decarboxylase
CLN8	CLN8 transmembrane ER and ERGIC protein	MSH2	mutS homolog 2
CNBP	CCHC-type zinc finger nucleic acid binding protein	MVP	major vault protein
CRABP1	cellular retinoic acid binding protein 1	NR1I2	nuclear receptor subfamily 1 group I member 2
CRABP2	cellular retinoic acid binding protein 2	NUDT19	nudix hydrolase 19
CRAT	carnitine O-acetyltransferase	PABPC1	poly(A) binding protein cytoplasmic 1
CTBP1	C-terminal binding protein 1	PEX11A	peroxisomal biogenesis factor 11 alpha
CTPS1	CTP synthase 1	PEX11B	peroxisomal biogenesis factor 11 beta
DHCR24	24-dehydrocholesterol reductase	PEX13	peroxisomal biogenesis factor 13
DHRS3	dehydrogenase/reductase 3	PEX14	peroxisomal biogenesis factor 14
DIO1	iodothyronine deiodinase 1	PEX2	peroxisomal biogenesis factor 2
DLG4	discs large MAGUK scaffold protein 4	PEX5	peroxisomal biogenesis factor 5
ECH1	enoyl-CoA hydratase 1	PEX6	peroxisomal biogenesis factor 6
ECI2	enoyl-CoA delta isomerase 2	PRDX1	peroxiredoxin 1
EHHADH	enoyl-CoA hydratase and 3-hydroxyacyl CoA dehydrogenase	PRDX5	peroxiredoxin 5
ELOVL5	ELOVL fatty acid elongase 5	RDH11	retinol dehydrogenase 11
RETSAT	retinol saturase	SLC35B2	solute carrier family 35 member B2

RXRG	retinoid X receptor gamma	SMARCC1	SWI/SNF related, matrix associated, actin depe regulator of chromatin subfamily c member 1
SCGB1A1	secretoglobin family 1A member 1	SOD1	superoxide dismutase 1
SCP2	sterol carrier protein 2	SOD2	superoxide dismutase 2
SEMA3C	semaphorin 3C	STS	steroid sulfatase
SERPINA6	serpin family A member 6	SULT2B1	sulfotransferase family 2B member 1
SIAH1	siah E3 ubiquitin protein ligase 1	TOP2A	DNA topoisomerase II alpha
SLC23A2	solute carrier family 23 member 2	TSPO	translocator protein
SLC25A17	solute carrier family 25 member 17	TTR	transthyretin
SLC25A19	solute carrier family 25 member 19	UGT2B17	UDP glucuronosyltransferase family 2 member
SLC25A4	solute carrier family 25 member 4	VPS4B	vacuolar protein sorting 4 homolog B
SLC27A2	solute carrier family 27 member 2	YWHAH	Tyrosine 3-monooxygenase/tryptophan monooxygenase activation protein eta

Supplementary Table 2. The 263 chemotherapy drugs approved by FDA or on clinical trials

Drug name	Drug name	Drug name
Curcumin	RH1	Chlorambucil
Chelerythrine	XK-469	Thiotepa
3-Bromopyruvate (acid)	5-fluoro deoxy uridine 10mer	Melphalan
Cordycepin	Seliciclib	Triethylenemelamine
Benzimate	Entinostat	Dromostanolone Propionate
Pimozide	Alvespimycin	Acrichine
Elesclomol	7-Tert-butyldimethylsilyl-10-hydroxycamptothecin	Fluorouracil
Wortmannin	Karenitecin	Nandrolone phenpropionate
geldanamycin analog	PX-316	Testolactone
Elliptinium Acetate	AFP464	Mithramycin
Triciribine phosphate	Rebimastat	Pipobroman
BEN	Imexon	Cyclophosphamide
Amonafide	E-7820	Mitomycin
Batracylin	LMP-400	Floxuridine
Buthionine sulphoximine	LMP776	Hydroxyurea
Tanespimycin	Lificguat	Uracil mustard
8-Chloro-adenosine	SR16157	Dexamethasone Decadron
Hypothemycin	Dimethylaminoparthenolide	Dacarbazine
Fostamatinib	Selumetinib	Dacarbazine
Pyrazoloacridine	BML-277	Vinblastine
Fenretinide	Obatoclax	Acetalax
Dolastatin 10	AT-13387	Cytarabine
Staurosporine	Itraconazole	Vincristine
Pyrazoloacridine	XL-147	Megestrol acetate
Lapachone	Hydrastinine HCl	tfdu
O-6-Benzylguanine	1st Precursor Intermediate to TDP 665759	Procarbazine
7-Hydroxystaurosporine	(+)-JQ1	Lomustine
7-Hydroxystaurosporine	Fenretinide	Daunorubicin
Perifosine	AP-26113	Daunorubicin
Alvocidib	By-Product of CUDC-305	Streptozocin
Midostaurin	LOR-253	Calusterone
XK-469	Pelitrexol	Estramustine
Triapine	Cobimetinib (isomer 1)	Vinblastine
kahalide f	Bafetinib	Fluphenazine
okadaic acid	Methotrexate	Arsenic trioxide
PD-98059	6-Mercaptopurine	Azacitidine
Epothilone B	6-Mercaptopurine	Cladribine
Aminoflavone	Nitrogen mustard	Mithramycin
BN-2629	Allopurinol	Asparaginase
LY-294002	Actinomycin D	Ifosfamide
Acetalax	Docetaxel	Pemetrexed
Fludarabine	Depsipeptide	Vismodegib
Cisplatin	Simvastatin	Actinomycin D
Isotretinoin	Raltitrexed	Mitomycin
Teniposide	7-Ethyl-10-hydroxycamptothecin	Lenvatinib
Doxorubicin	Bortezomib	Nelarabine
Fludarabine	Irofulven	Crizotinib
Bleomycin	Temsirolimus	Daunorubicin
Paclitaxel	Denileukin Diftitox Ontak	Digoxin
Decitabine	Pemetrexed	Ethinyl estradiol
Mitomycin	Vorinostat	Fluorouracil
Bendamustine	Estramustine	Nitrogen mustard
Etoposide	Arsenic trioxide	Melphalan
Homoharringtonine	Eribulin mesilate	6-Mercaptopurine
Mithramycin	Gefitinib	Tyrothricin
Tegafur	Erlotinib	Vinblastine

Parthenolide	Fulvestrant	Cabozantinib
Dexrazoxane	Celecoxib	Axitinib
Tamoxifen	Zoledronate	Etoposide
Pentostatin	Belinostat	Azacitidine
Rapamycin	Lapatinib	Floxuridine
Carboplatin	Irinotecan	Trametinib
Valrubicin	Dasatinib	Palbociclib
Idarubicin	Everolimus	Carfilzomib
Epirubicin	Pazopanib	Homoharringtonine
Oxaliplatin	Imatinib	Ixazomib citrate
Mitoxantrone	Lapatinib	Teniposide
Cytarabine	Nelfinavir	Ponatinib
Mitoxantrone	Nilotinib	Bleomycin
Fludarabine	Olaparib	Paclitaxel
Imiquimod	Ixabepilone	Rapamycin
Carmustine	Raloxifene	Teniposide
Mithramycin	Abiraterone	Simvastatin
Rapamycin	Abiraterone	Belinostat
Clofarabine	Sunitinib	Doxorubicin
Vinorelbine	Afatinib	Vincristine
Topotecan	Pazopanib	Pipamperone
Gemcitabine	Olaparib	Epirubicin
bisacodyl, active ingredient of viraplex	Depsipeptide	Idelalisib
Irinotecan	Pralatrexate	Topotecan
Arsenic trioxide	Vinorelbine	Dabrafenib
6-Mercaptopurine	Vandetanib	Bosutinib
Docetaxel	Cabozantinib	ABT-199
Vorinostat	Panobinostat	LDK-378
Gefitinib	Sonidegib	LDK-378
Clofarabine	Vemurafenib	AZD-9291
Dasatinib	Ibrutinib	Dabrafenib
Irinotecan	Alectinib	

Table S3. The detail information of the primer sequences used in real-time quantitative-polymerase chain reaction

Gene	Forward Primer	Reverse Primer
ABCC5	AGAAGTGTGAGGGAGAGAACCAG	GGTGCTGGTGTTTGGAAGTAGT
BCL10	TCTGAAAGATGGAGCCACGAA	CGTGCTGGATTCTCCTTCTGG
FDPS	AGCAGCCCTATTACCTGAACC	CATCCTGAATCTGAAAGAACTCCC
ITGB1BP1	TGTTACGATGACGGTCTGGG	CAAATGGCTTGTGCTTGTTCC
MSH2	GTGAGTCAGCAGAAGTGTCCA	CCCAAATCCATCGTAGGTAGAAGT
PABPC1	AAGGTTATGATGGAGGGTGGTC	GGGTTGATTACAGGGTTGGGAA
PRDX1	ACTGTAAATGACCTCCCTGTTGG	CTTTGCTCTTTTGGACATCAGGC
SLC25A19	AATGAGAACCTCCAAAACCTGC	CTCAAACCCTCCAACCTGTAGC
YWHAH	AGCAGCATTGAGCAGAAAACC	CACCTTGCTCTCATACTGGAAATC
beta-actin	CACCCAGCACAATGAAGATCAAGAT	CCAGTTTTTAAATCCTGAGTCAAGC

Table S4. The detail information of the antibodies used in immunohistochemistry

Gene	Antibody Name	Antibody Number	Company
ABCC5	MRP5 Polyclonal Antibody	19503-1-AP	Proteintech
BCL10	Anti-Bcl10 antibody [SN74-04]	ET1611-79	HUABIO
FDPS	FDPS Polyclonal Antibody	16129-1-AP	Proteintech
ITGB1BP1	ITGB1BP1 Polyclonal Antibody	12300-1-AP	Proteintech
MSH2	Anti-MSH2 antibody [10G1]	EM1801-04	HUABIO
PABPC1	Anti-PABP antibody	ER1919-24	HUABIO
PRDX1	Anti-Peroxiredoxin 1 antibody [JF0945]	ET1702-08	HUABIO
SLC25A19	Rabbit Anti-SLC25A19 antibody (bs-21222R)	bs-21222R	BIOSS
YWHAH	Anti-14-3-3 η antibody	ER62526	HUABIO

Supplementary Table 5. Correlation analysis shows the relationship of the prognostic genes with chemotherapy drug sensitivity

Gene	Drug	correlation coefficient	P value
SLC25A19	Vorinostat	0.488648	7.46E-05
SLC25A19	Hydroxyurea	0.486019	8.26E-05
ABCC5	Vorinostat	0.470842	0.000147
SLC25A19	Cladribine	0.450555	0.000303
SLC25A19	Parthenolide	0.448784	0.000323
SLC25A19	Dromostanolone Propionate	0.432741	0.000554
PRDX1	Vemurafenib	-0.4266	0.000676
ABCC5	Nelarabine	0.422364	0.000775
SLC25A19	Cytarabine	0.418325	0.000881
PRDX1	Dabrafenib	-0.41739	0.000907
SLC25A19	Methotrexate	0.407647	0.001226
SLC25A19	Chelerythrine	0.404045	0.001367
SLC25A19	Fludarabine	0.398597	0.001608
SLC25A19	Imexon	0.396318	0.00172
SLC25A19	Fostamatinib	0.392355	0.001931
SLC25A19	Cyclophosphamide	0.391242	0.001995
MSH2	Nelarabine	0.389333	0.002108
SLC25A19	Carmustine	0.387178	0.002242
ABCC5	Fludarabine	0.384259	0.002436
SLC25A19	Chlorambucil	0.38155	0.00263
SLC25A19	Belinostat	0.380493	0.002709
SLC25A19	Pipobroman	0.379926	0.002752
MSH2	Chelerythrine	0.37854	0.002861
SLC25A19	6-Mercaptopurine	0.370905	0.003529
FDPS	Hydroxyurea	0.368924	0.003724
SLC25A19	Uracil mustard	0.367938	0.003824
SLC25A19	Triethylenemelamine	0.361946	0.004488
MSH2	PX-316	0.36027	0.00469
SLC25A19	Thiotepa	0.357251	0.005076
SLC25A19	RH1	0.356957	0.005115
SLC25A19	8-Chloro-adenosine	0.354962	0.005387
SLC25A19	Fenretinide	0.350911	0.005978
ABCC5	PX-316	0.350404	0.006056
YWHAH	Valrubicin	-0.34959	0.006183
ABCC5	Chelerythrine	0.347579	0.006506
SLC25A19	AT-13387	0.343111	0.007279
SLC25A19	Raltitrexed	0.342894	0.007318
ITGB1BP1	Chelerythrine	0.341681	0.007542
SLC25A19	Lomustine	0.341282	0.007617
SLC25A19	Belinostat	0.380493	0.002709
MSH2	Everolimus	-0.34082	0.007704
PRDX1	Selumetinib	-0.33913	0.008034
SLC25A19	Acrichine	0.33754	0.008353
SLC25A19	Melphalan	0.336665	0.008533
ABCC5	Cladribine	0.336261	0.008618
SLC25A19	Dasatinib	-0.33566	0.008745
YWHAH	Actinomycin D	-0.33465	0.008963
PRDX1	okadaic acid	-0.33195	0.009567
SLC25A19	5-fluoro deoxy uridine 10mer	0.328747	0.010329
SLC25A19	kahalide f	-0.32777	0.010572
FDPS	Nelarabine	0.326977	0.010773
SLC25A19	Asparaginase	0.326437	0.010911
BCL10	Fluorouracil	-0.32632	0.010941
SLC25A19	Lenvatinib	-0.32516	0.011244
MSH2	Amonafide	0.324716	0.011363

MSH2	Allopurinol	0.324426	0.011441
FDPS	Fenretinide	0.323971	0.011564
FDPS	Paclitaxel	-0.32385	0.011598
FDPS	Dimethylaminoparthenolide	0.323055	0.011814
MSH2	3-Bromopyruvate (acid)	0.322832	0.011876
SLC25A19	Pralatrexate	0.322051	0.012095
FDPS	Chelerythrine	0.321831	0.012157
SLC25A19	Vismodegib	0.320939	0.012412
FDPS	Chlorambucil	0.319188	0.012926
YWHAH	Depsipeptide	-0.31606	0.01389
MSH2	Pyrazoloacridine	0.313164	0.014836
YWHAH	Mithramycin	-0.31316	0.014838
SLC25A19	Nelarabine	0.312266	0.015141
BCL10	Lenvatinib	0.311226	0.0155
PABPC1	Homoharringtonine	-0.31003	0.015924
ABCC5	Testolactone	0.309677	0.016049
SLC25A19	Ixabepilone	0.309172	0.016231
BCL10	Simvastatin	0.309028	0.016284
SLC25A19	Staurosporine	-0.30681	0.017109
SLC25A19	Allopurinol	0.306623	0.01718
SLC25A19	Entinostat	0.304859	0.017864
PRDX1	Digoxin	0.303485	0.018412
PRDX1	Perifosine	0.302544	0.018796
ABCC5	Dolastatin 10	-0.30042	0.019689
ITGB1BP1	Asparaginase	0.300376	0.019707
SLC25A19	Carboplatin	0.300232	0.019768
MSH2	Dexamethasone Decadron	0.300046	0.019848
SLC25A19	Calusterone	0.298571	0.020493
MSH2	Fenretinide	0.298294	0.020616
SLC25A19	Lapachone	0.297983	0.020755
SLC25A19	LMP-400	0.297398	0.021018
PRDX1	Cobimetinib (isomer 1)	-0.29721	0.021101
PRDX1	Bafetinib	-0.29712	0.021144
BCL10	Tegafur	-0.29689	0.02125
PRDX1	Midostaurin	0.296522	0.021418
ITGB1BP1	Fluorouracil	-0.2964	0.021475
MSH2	Vorinostat	0.29547	0.021906
FDPS	LMP-400	0.294803	0.022221
SLC25A19	Benzimate	0.294689	0.022275
MSH2	Parthenolide	0.293895	0.022655
BCL10	Tanespimycin	-0.29364	0.022777
PRDX1	Pipamperone	-0.29352	0.022834
YWHAH	Entinostat	-0.29345	0.022869
BCL10	Vemurafenib	-0.29226	0.023454
SLC25A19	Irinotecan	0.292164	0.023503
YWHAH	Epirubicin	-0.29192	0.023624
SLC25A19	Nitrogen mustard	0.291167	0.024003
SLC25A19	Raloxifene	0.291136	0.024019
PRDX1	Ethinyl estradiol	-0.29065	0.024269
FDPS	Uracil mustard	0.290032	0.024584
PABPC1	Depsipeptide	-0.28972	0.024747
SLC25A19	XK-469	0.287565	0.025887
FDPS	kahalide f	-0.28714	0.026115
FDPS	Cyclophosphamide	0.287038	0.026173
FDPS	Vinblastine	-0.28693	0.026231
ITGB1BP1	Amonafide	0.286701	0.026357
FDPS	Parthenolide	0.285461	0.027044
YWHAH	Lificguat	-0.28531	0.027128
SLC25A19	Dimethylaminoparthenolide	0.284862	0.027381
PABPC1	Doxorubicin	-0.28452	0.027576

FDPS	Actinomycin D	-0.28432	0.02769
SLC25A19	Ifosfamide	0.283113	0.028386
FDPS	okadaic acid	-0.27983	0.030356
ABCC5	Dasatinib	-0.27934	0.030656
ABCC5	Fenretinide	0.278899	0.030935
YWHAH	Doxorubicin	-0.27839	0.031257
PRDX1	Eribulin mesilate	-0.27816	0.031399
FDPS	Depsipeptide	-0.27782	0.031614
MSH2	Zoledronate	-0.27742	0.031871
SLC25A19	tfdu	0.27741	0.031879
PRDX1	Mitoxantrone	-0.2773	0.031947
SLC25A19	Clofarabine	0.276153	0.032696
MSH2	Fludarabine	0.275498	0.033128
ITGB1BP1	Hydroxyurea	0.275461	0.033152
FDPS	Dolastatin 10	0.2752	0.033327
BCL10	Dabrafenib	-0.27502	0.033447
YWHAH	Eribulin mesilate	-0.27374	0.034311
SLC25A19	Oxaliplatin	-0.2734	0.034546
FDPS	Mithramycin	0.273192	0.034689
MSH2	AT-13387	-0.27318	0.034693
PABPC1	Paclitaxel	0.273042	0.034792
MSH2	Olaparib	-0.27233	0.035286
ABCC5	3-Bromopyruvate (acid)	-0.27158	0.035815
ABCC5	Chlorambucil	0.270527	0.036567
PABPC1	Denileukin Diftitox Ontak	0.27012	0.036861
FDPS	Dexamethasone Decadron	-0.26983	0.037071
YWHAH	Paclitaxel	0.26982	0.037079
FDPS	Melphalan	-0.26972	0.037149
PABPC1	Elliptinium Acetate	0.268993	0.037686
PABPC1	okadaic acid	-0.2686	0.037975
YWHAH	Teniposide	-0.26855	0.038012
SLC25A19	Buthionine sulphoximine	0.268482	0.038039
BCL10	Panobinostat	-0.26826	0.038065
MSH2	Ifosfamide	0.268004	0.038229
ITGB1BP1	Acetalax	-0.26776	0.038422
PABPC1	Vinorelbine	-0.26748	0.038607
BCL10	Tamoxifen	-0.26736	0.038816
ABCC5	kahalide f	-0.26731	0.038905
PRDX1	Hypothemycin	-0.26556	0.038946
SLC25A19	LMP776	0.265251	0.040296
FDPS	Triethylenemelamine	0.265109	0.040535
ITGB1BP1	bisacodyl, active ingredient of viraplex	-0.26464	0.040647
FDPS	Thiotepa	0.264496	0.041021
BCL10	Midostaurin	0.263963	0.041131
SLC25A19	Testolactone	0.263816	0.041556
BCL10	Denileukin Diftitox Ontak	-0.26349	0.041939
SLC25A19	Nelfinavir	0.263467	0.041955
ITGB1BP1	Ifosfamide	0.263313	0.042079
PRDX1	Nitrogen mustard	0.263292	0.042096
FDPS	Tanespimycin	-0.26268	0.042591
YWHAH	Homoharringtonine	-0.26257	0.042688
PABPC1	Allopurinol	0.262241	0.042954
PABPC1	Fludarabine	0.262048	0.043113
FDPS	Vincristine	-0.26119	0.043826
PRDX1	Hydroxyurea	0.26002	0.044814
SLC25A19	Imatinib	0.259402	0.045344
FDPS	LMP776	0.259184	0.045531
SLC25A19	Curcumin	0.258895	0.045782
FDPS	Homoharringtonine	-0.25889	0.045783
ITGB1BP1	Vorinostat	0.258781	0.04588

MSH2	O-6-Benzylguanine	-0.25859	0.046047
PRDX1	Irofulven	0.258234	0.046357
SLC25A19	XL-147	0.257094	0.047365
PRDX1	Dexrazoxane	0.256797	0.047631
ITGB1BP1	Everolimus	-0.25639	0.047992
ITGB1BP1	Elesclomol	-0.25636	0.048021
FDPS	Panobinostat	-0.25635	0.048029
SLC25A19	Fluphenazine	0.255935	0.048408
PRDX1	Idarubicin	0.255365	0.048928
FDPS	Imexon	0.255034	0.049232
BCL10	Staurosporine	0.254484	0.04974
